# Motor neuron disease can present as a paraneoplastic neurologic syndrome with various phenotypes

**DOI:** 10.1093/braincomms/fcag024

**Published:** 2026-03-09

**Authors:** Eleftheria Koropouli, Stavros Bellos, Stavroula Aristeidou, Ariadne Daponte, Panagiotis Gklinos, Fotios Athanasopoulos, Antonis Tsionis, Elisabeth Andreadou, Vasiliki Zouvelou, Michail Rentzos

**Affiliations:** First Department of Neurology, Aiginition Hospital, National and Kapodistrian University of Athens School of Medicine, Vasilissis Sofias Avenue 72-74, Athens 11528, Greece; First Department of Neurology, Aiginition Hospital, National and Kapodistrian University of Athens School of Medicine, Vasilissis Sofias Avenue 72-74, Athens 11528, Greece; First Department of Neurology, Aiginition Hospital, National and Kapodistrian University of Athens School of Medicine, Vasilissis Sofias Avenue 72-74, Athens 11528, Greece; First Department of Neurology, Aiginition Hospital, National and Kapodistrian University of Athens School of Medicine, Vasilissis Sofias Avenue 72-74, Athens 11528, Greece; First Department of Neurology, Aiginition Hospital, National and Kapodistrian University of Athens School of Medicine, Vasilissis Sofias Avenue 72-74, Athens 11528, Greece; First Department of Neurology, Aiginition Hospital, National and Kapodistrian University of Athens School of Medicine, Vasilissis Sofias Avenue 72-74, Athens 11528, Greece; First Department of Neurology, Aiginition Hospital, National and Kapodistrian University of Athens School of Medicine, Vasilissis Sofias Avenue 72-74, Athens 11528, Greece; First Department of Neurology, Aiginition Hospital, National and Kapodistrian University of Athens School of Medicine, Vasilissis Sofias Avenue 72-74, Athens 11528, Greece; First Department of Neurology, Aiginition Hospital, National and Kapodistrian University of Athens School of Medicine, Vasilissis Sofias Avenue 72-74, Athens 11528, Greece; First Department of Neurology, Aiginition Hospital, National and Kapodistrian University of Athens School of Medicine, Vasilissis Sofias Avenue 72-74, Athens 11528, Greece

**Keywords:** motor neuron disease, amyotrophic lateral sclerosis, paraneoplastic neurologic syndrome, cancer

## Abstract

Paraneoplastic motor neuron disease is an uncommon paraneoplastic neurologic syndrome whose existence has fallen into ambiguity. Epidemiologic studies that have addressed the association between cancer and motor neuron disease have provided conflicting results. Case studies that report motor neuron disease presentation at the time of active malignant disease, in the presence of another paraneoplastic neurologic syndrome or onconeural antibody or with neurologic response to antineoplastic treatment provide strong evidence for paraneoplastic motor neuron disease. However, conclusive evidence about the existence and the clinical and laboratory profiles of this neurologic syndrome is lacking. In this study, we report four new cases of paraneoplastic motor neuron disease, two of whom with expression of Sry-like high mobility group box 1 (SOX1) antibody. We also present a systematic review of all cases of paraneoplastic motor neuron disease reported to date that fulfill prespecified inclusion criteria with individual participant data meta-analysis of the demographic, clinical and laboratory features of the disease. Our data demonstrate that motor neuron disease can present as a paraneoplastic neurologic syndrome. Paraneoplastic motor neuron disease spans the whole motor neuron disease phenotypic spectrum, and it is associated with a wide variety of neoplastic diseases, onconeural antibodies and it may present concurrently with other well-recognized paraneoplastic neurologic syndromes. Paraneoplastic motor neuron disease may be clinically indistinguishable from idiopathic motor neuron disease. Its only distinctive clinical feature is the rapidly progressive course. A subset of cases display immune derangements in cerebrospinal fluid, including increased white cell count, elevated protein, albumin index, IgG index and/or oligoclonal band expression. Cancer-induced inflammatory pathways may trigger the disease in genetically predisposed individuals harboring amyotrophic lateral sclerosis-causing genetic deficits. A thorough evaluation for neoplastic diseases should be carried out upon strong suspicion of this rare paraneoplastic neurologic syndrome to increase the diagnostic yield for this entity. Paraneoplastic motor neuron disease apparently results from complex interactions between degenerative and immune pathways and its pathophysiology may elucidate previously unresolved aspects of idiopathic motor neuron disease pathogenesis.

## Teaser

Koropouli *et al*. report a dataset of 163 subjects with paraneoplastic motor neuron disease, demonstrating that motor neuron disease may be of paraneoplastic origin, may present in association with various neoplasms, onconeural antibodies and concomitantly with other paraneoplastic neurologic syndromes and may respond to cancer treatment providing proof of concept.

## Graphical Abstract

**Graphical Abstract:**
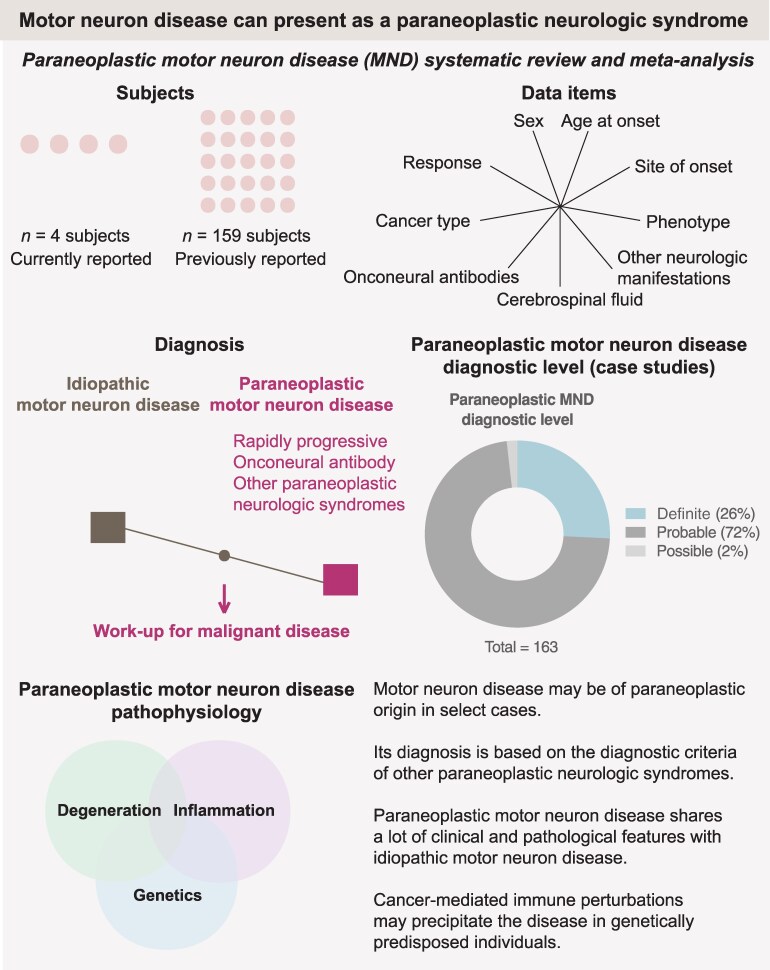

## Introduction

Motor neuron disease (MND) is an invariably progressive degenerative disorder of the nervous system that primarily affects motor neurons, including upper motor neurons (corticospinal, corticobulbar) and lower motor neurons (spinal, bulbar). These topographically ordered motor neurons can be affected in any combination in time and in space giving rise to various MND phenotypes including amyotrophic lateral sclerosis (ALS), primary lateral sclerosis (PLS), progressive bulbar palsy (PBP) and progressive muscular atrophy (PMA), depending on the spatiotemporal pattern of their dysfunction. It has long been recognized that certain factors may underlie MND in isolated cases, giving rise to secondary or as originally called ‘symptomatic’ MND,^1^ including paraneoplastic MND.^2^ Paraneoplastic neurologic syndromes (PNS) are immune-mediated neurologic disorders triggered by occult or clinically evident cancer.^3,4^ In several cases, however, in lack of a close temporal link to cancer diagnosis or of evidence of response to immune or antineoplastic therapy, the paraneoplastic nature of MND has been disputed.^5-9^ This has led to a flailing concept of paraneoplastic MND, which either is classified as a ‘non-classical’ PNS or it is not addressed at all.^10,11^

To systematically explore paraneoplastic MND, we pursued a critical and thorough review of all reported paraneoplastic MND case studies, with meta-analysis of demographic, clinical and laboratory disease parameters on a patient-by-patient basis. In addition, we report four cases of paraneoplastic MND evaluated at our clinic. Based on the results of meta-analysis, we formulate diagnostic recommendations for paraneoplastic MND that may inform clinical practice and aid in decision-making for paraneoplastic MND evaluation. Finally, we elaborate on MND neuropathology and unfold the continuum of MND spectrum disorders.

## Materials and methods

### Cases

The four cases of paraneoplastic MND reported here were evaluated at the MND outpatient center and the Division of Neuromuscular Diseases of Aiginition Hospital in Athens, Greece, between January 2020 and December 2024. The patients were evaluated with established diagnostic approaches and treated in agreement with international consensus guidelines and in accordance with institutional policies and ethics.

### Systematic review and individual participant data meta-analysis

#### Search strategy

We performed electronic search of PubMed/Medline databases and also free electronic search for all articles published on paraneoplastic MND from 1954 when the first case was reported, up to 31 December 2024. Search terms included ‘paraneoplastic motor neuron disease’, ‘paraneoplastic amyotrophic lateral sclerosis’, ‘motor neuron disease and cancer’, ‘motor neuron disease and malignant disease’, ‘paraneoplastic neurologic syndromes’, ‘motor neuron disease and lymphoproliferative disorders’. We also reviewed the bibliographies of retrieved articles. The review was carried out manually and presented according to the preferred reporting items for systematic reviews and meta-analyses (PRISMA) guidelines.^12^ Automation tools were not used for either article selection or data extraction.

#### Selection process, inclusion and exclusion criteria

Our search yielded 155 case studies, which were evaluated for their relevance and strength of evidence of MND and paraneoplastic MND. Article selection was done by two authors (E.K., S.B.). MND cases were evaluated for their paraneoplastic origin according to the updated diagnostic criteria for PNS.^4^ MND cases were considered paraneoplastic if they gathered ≥ 4 points in the PNS-Care scoring system, with the following assumptions adopted for MND for the purpose of our analysis: (i) MND is an intermediate-risk phenotype, (ii) The co-occurrence of MND and a high-risk phenotype is a high-risk phenotype, (iii) Detection of neoplastic disease is scored regardless of the type of neoplasm. The time cut-off for a close temporal association of MND with cancer is considered the 2-year period, with the exception of 8 cases who have had a distal temporal relation (> 2 years) of MND onset with cancer diagnosis but had other strong evidence for paraneoplastic MND, e.g. onconeural antibody expression, neurologic response to tumor treatment, which also includes one individual described in this study (case 2).^6,13-18^ With the application of these criteria, the review and meta-analysis include 90 case studies (89 from literature and this study) and 163 patients (159 from literature and four cases reported here) (Supplementary Table 1). All cases of definite, probable and possible paraneoplastic MND are included (Supplementary Table 2). Among all cases, 26% (42 of 163) fulfill the requirements for definite paraneoplastic MND, 72% (118 of 163) fulfill the requirements for probable paraneoplastic MND and 2% (3 of 163) fulfill the requirements for possible paraneoplastic MND. In several cases there is response to antineoplastic and/or immune therapy, which is not scored in this scale, and this is reported as a non-scorable parameter in Supplementary Table 2. In cases with MND and lymphoproliferative disorders onconeural antibodies are not tested and PNS-Care score is low. Exclusion criteria include poorly documented MND, no cancer verified along with absence of additional disease-specific features, motor neuropathy, monoclonal gammopathies of undetermined significance (MGUS), suspected radiation myelopathy and malignant spread into the CNS. Of all articles included, five are written in non-English language and their full text was not found but their abstracts are available in English,^19-23^ three are written in non-English language but their full text was found and translated^24-26^ and one is published in English as abstract only.^27^

#### Data collection

Data were independently collected and analyzed by E.K. and S.B., three times by each rater to ensure intra-rater and inter-rater consistency. The collected data items (variables of interest) include patient’s sex, age at MND onset (years), site of MND onset, MND phenotype, neoplastic disease, temporal association of MND with neoplastic disease, onconeural and other antibodies, cerebrospinal fluid (CSF) analysis, therapeutic interventions and outcome. The patient’s sex refers to the sex assigned at birth (biological). Not all data items are reported for all patients; for each item, the total number of patients for whom information is available is shown at the bottom of graph.

#### Statistical analysis

Data collection, analysis, statistics and graph generation are performed in GraphPad Prism. Data are presented in parts of whole (circle) graphs where color shades represent mutually exclusive categories whose sum equals to 100%, or in column graphs. In parts of whole graphs, subgroups are shown with their percentage of occurrence on the side of the graph, whereas sample size (*n*) is reported in figure legend. In these datasets, there is no further comparison between subgroups and no further statistical analysis. Description on the analysis of each dataset is provided in figure legend.

#### Site of onset

The clinical site of MND onset allows the inference of the pathological site of disease onset in case of pure lower motor neuron involvement, since autopsy studies have shown pathologic perturbations at spinal levels that account for symptoms, e.g. a patient with lower limb onset has prominent motor neuron loss in lumbar spinal cord.^28^ Unlike pure lower MND, if upper motor neurons are also involved, the pathological site of onset cannot be inferred from the symptomatic bodily region. To avoid clinical-pathological discrepancies, the site of onset reported here is the clinical site of onset (symptomatic bodily region).

#### Ethics statement

All four MND-affected individuals presented here have given informed written consent according to the Declaration of Helsinki. The study has been approved by the Institutional Review Board and the Institution’s Ethics Committee.

## Results

### Case 1

A 51-year-old woman presented with weakness of the right upper limb of subacute onset. One year later she deteriorated with weakness of upper and lower limbs bilaterally, dysarthria and dysphagia. Neurologic evaluation revealed pyramidal, lower motor neuron, bulbar and pseudobulbar signs. Brain and spinal cord magnetic resonance imaging was unremarkable. Nerve conduction studies showed lowered amplitude of motor nerve compound muscle action potentials, and needle electrode electromyography demonstrated fibrillation potentials and positive sharp waves at three spinal levels and in the tongue. The diagnosis of ALS was made. Rapid neurologic progression prompted a work-up for neoplastic diseases. Chest and abdomen computed tomography (CT) was unrevealing, whereas mammography revealed a breast tumor that was excised and demonstrated to be invasive ductal carcinoma. The patient received local radiotherapy and was placed on tamoxifen. During radiotherapy, oral methylprednisolone led to neurologic improvement, whereas limb weakness re-emerged upon steroid tapering. CSF analysis and cytology were unrevealing (white blood cells, 0; protein, 22 mg/dL; albumin index, 4.54; IgG index, 0.53; no oligoclonal bands). Onconeural antibody testing in serum was negative. Two five-day courses of intravenous methylprednisolone 3 months apart, 5 gr each, followed by two courses of intravenous immunoglobulin (IVIG) 3 weeks apart, each 2 gr/kg over 5 days, led to neurologic stabilization. For long-term control the patient was placed on rituximab. Follow-up evaluations were negative for residual malignant disease or recurrence. However, the patient presented deep vein thrombosis with pulmonary embolism and passed away 2 years and 7 months after ALS onset.

### Case 2

A 74-year-old man presented with a 3-year history of left lower limb weakness, whereas a few years later he developed dysarthria and difficulty swallowing. At the time of evaluation, he was wheelchair-bound and had significant weight loss. Neurologic examination revealed dysarthria, dysphagia, severe lower limb weakness, pyramidal syndrome bilaterally, severe weakness and atrophy of first dorsal interosseous and thenar muscles bilaterally, dysmetria and dysdiadochokinesia. Magnetic resonance imaging of the brain and spinal cord was unremarkable. Nerve conduction studies were insignificant. Needle electrode electromyography demonstrated evidence of active and chronic denervation in lower and upper limbs. Of note, ALS symptoms aggravated the last few months before presentation. We made the diagnosis of ALS with accompanying cerebellar degeneration. CT of the chest revealed a mass in the right lung with infiltration of the regional lymph nodes, which turned out to be squamous lung carcinoma. CSF analysis showed increased protein and albumin index (white blood cells, 0; protein, 67 mg/dL; albumin index, 16.61; IgG index, 0.40; no oligoclonal bands). Onconeural antibody testing was positive for expression of the Sry-like high mobility group box 1 (SOX1) antibody. In this case, MND prevailed in the clinical picture, whereas cerebellar degeneration was subclinical with cerebellar signs only on examination. Further, MND had an indolent onset, remained stable for a few years that prevented early diagnosis, and it progressed later when diagnosed concurrently with cerebellar degeneration. The constellation of ALS and cerebellar degeneration along with SOX1 antibody expression and lung cancer detection established the diagnosis of paraneoplastic ALS. The patient denied antineoplastic or immune therapy and died 4 years after MND symptom onset.

### Case 3

A 63-year-old man presented with lower limb weakness followed shortly by upper limb weakness. Five months later he presented dysarthria, followed by difficulty swallowing. Over the last 6 months he had significant weight loss and painful muscle cramps particularly when walking. His history was notable for urinary bladder cancer diagnosed and treated with cystectomy 1 year earlier. Neurologic examination revealed pseudobulbar speech, left-sided pyramidal syndrome, severe weakness, atrophy and fasciculations in upper and lower limbs and the tongue. MRI of the brain and spinal cord was unremarkable. CSF analysis was unrevealing (white blood cells, 0; protein, 23 mg/dL; albumin index, 3.52; IgG index, 0.41; no oligoclonal bands). Nerve conduction studies were unrevealing, whereas needle electrode electromyography demonstrated fibrillation potentials, positive sharp waves, and polyphasic motor unit action potentials with high amplitude and long duration in upper and lower limb muscles. These findings established the diagnosis of ALS. Due to rapid neurological deterioration and the history of cancer, we pursued onconeural antibody testing that revealed robust SOX1 antibody expression, confirmed in two different laboratories. Chest and abdomen CT and whole-body PET-CT were unrevealing. Genetic testing for *C9orf72* turned out negative. The patient received a 5-day course of IVIG followed by a 3-day course of intravenous methylprednisolone, yet neither elicited a clinical response. One month later treatment with cyclophosphamide was initiated but his condition continued to deteriorate. Despite the absence of clinical response, subsequent testing for SOX1 antibodies revealed reduced antibody titers, which can be attributed to the strong immunosuppressive regimens. The rapidly progressive course of ALS, the recent history of cancer and the strong SOX1 antibody expression make the diagnosis of paraneoplastic ALS, despite the absence of response to immune treatments. Lack of response cannot rule out the paraneoplastic nature of the disease since most PNS display a progressive course despite treatment.^29,30^

### Case 4

A 73-year-old woman presented with a 22-month history of dysarthria, a 17-month history of gait unsteadiness, while the last 7 months she had difficulty swallowing, orthostatic hypotension and dropped head. Neurologic examination revealed bulbar dysarthria, difficulty swallowing, limb weakness, pyramidal syndrome, prominent cerebellar dysfunction with inability to perform tandem gait, dysdiadochokinesia, and hypermetria on saccades and on finger chase and extremely severe autonomic failure that completely prevented her from standing up because of orthostatic hypotension. Magnetic resonance imaging of the brain and cervical spine was unremarkable. Needle electromyography revealed active denervation in lower limbs and chronic denervation in upper and lower limbs. CSF analysis was unrevealing (cells, 0; protein, 21 mg/dL; albumin index, 2.68; IgG index, 0.58; no oligoclonal bands, cytology negative for cancer). The clinical and laboratory findings established the diagnosis of ALS, cerebellar dysfunction and autonomic failure, which prompted a work-up for neoplastic diseases. Onconeural antibody testing turned out negative both in cell-based assays and on cerebellar tissue. CT of the chest revealed a solid lung nodule with speculated borders in the left lower lobe, which was hypermetabolic on PET-CT, compatible with lung cancer. A 5-day course of IVIG led to neurologic improvement with significant amelioration of dysphagia, dysarthria and cerebellar symptoms. Two months later a second 5-day course of IVIG was administered, followed by surgical excision of the tumor. Histologic examination and immune phenotyping of the neoplastic nodule showed neuroendocrine small cell lung cancer. Following surgery, the patient was intubated and hospitalized in the intensive care unit. At this time, the patient breaths spontaneously, she has feeding gastrostomy but she also eats by mouth, she speaks with mild dysarthria and ambulates with support. Autonomic failure and cerebellar symptoms have regressed to a great extent, and MND symptoms have been ameliorated. The neuroendocrine nature of the tumor, the parallel progression of different neurologic syndromes and neurologic response to immune and cancer treatments point toward MND of paraneoplastic origin in this patient.

### Motor neuron disease can present as a paraneoplastic neurologic syndrome

Paraneoplastic MND has been a subject of controversy that has been tackled by epidemiologic studies and case studies, with opposing views.^31^ Epidemiologic studies have attempted to explore an association between cancer and MND by comparing cohorts of patients with cohorts of healthy individuals. The incidence of cancer among MND patients was calculated at 10%, which is significantly higherthan the 1.6% calculated for the control cohort,^32^ in accordance with more recent studies showing a higher-than-expected co-incidence of the two entities that may result from a causal relationship between the two diseases.^18,33^ Other epidemiologic studies have, however, failed to show a rate of co-occurrence that exceeds that of the general population.^32,34,35^ Overall, epidemiologic studies have not provided conclusive evidence and have resulted in uncertainties. However, these studies are confounded by the rarity of MND, the lack of data that can prove causality and possible missing of diagnosis in retrospective registry-based studies. On the other hand, there is an ever-growing list of case studies that report MND evolving concomitantly with a neoplastic disease. Whether in these cases MND is a PNS or solely a co-incidence is answered by cases in which cancer presents concurrently with MND and another more common PNS, with onconeural antibody expression, with or without response to immune or antineoplastic treatment.^24,36-40^ In the four cases presented here, MND presented or significantly aggravated at the time of cancer manifestation, in some cases in parallel with other PNS and in two of them MND symptoms stabilized after tumor treatment, features rather compatible with paraneoplastic MND. MND may also present with non-neurologic paraneoplastic syndromes including Sweet’s syndrome or acanthosis nigricans.^19,41^ In isolated cases, MND remission has been associated with a decrease in onconeural antibody titers,^42^ implying an association of MND with underlying immune perturbations. Further, immune checkpoint inhibitors have been related to MND progression.^43^ Additional evidence for paraneoplastic MND is derived from studies of cancer patients whose autopsy revealed marked loss of motor neurons in spinal cord ventral horns.^44^ Taken together, these data strongly support the paraneoplastic origin of MND in select cases.

### Systematic review and meta-analysis

**Figure 1:**
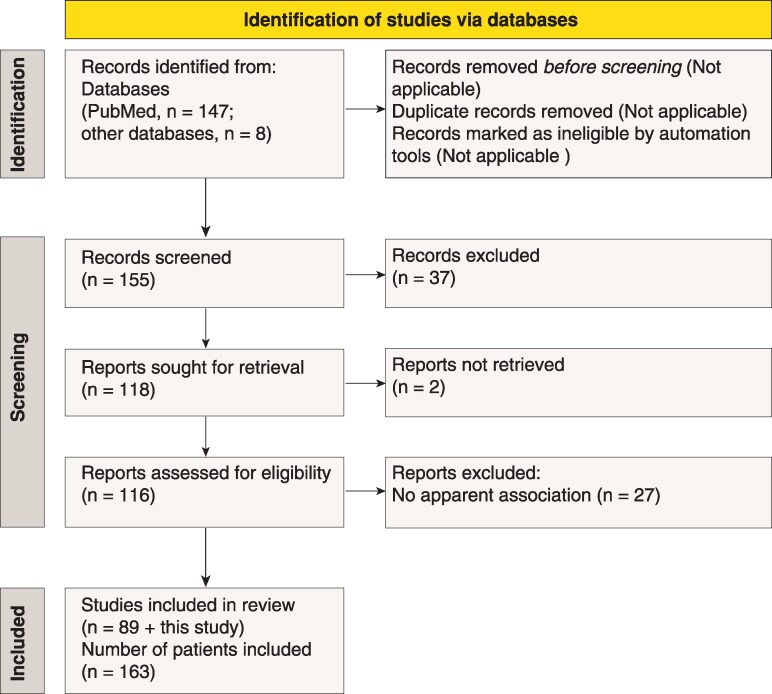
**Workflow of screening, identification and selection of paraneoplastic MND case studies.** Graphic overview of the processes of screening, identification and selection of case studies of paraneoplastic MND, reported according to the PRISMA guidelines. The diagnostic, inclusion and exclusion criteria applied for study selection are outlined in Materials and Methods. In total, 90 case studies and 163 human subjects are included, which encompass the present study with the four subjects diagnosed with paraneoplastic MND.

**Figure 2:**
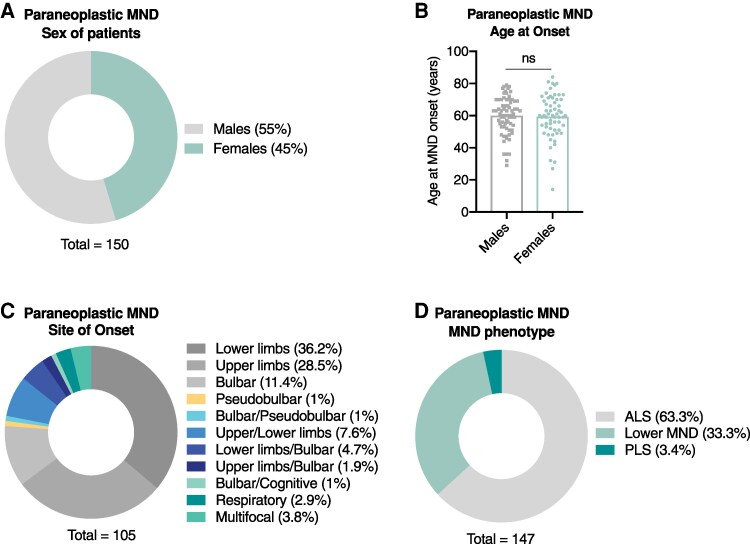
**Demographic and clinical characteristics of the paraneoplastic MND cohort.** (**A**) Sex of paraneoplastic MND patients. There is a slight preponderance of males (*n* = 82) compared with females (*n* = 68), with a male-to-female ratio 1.2. For 13 out of 163 patients the sex is not reported. (**B**) Age at onset of paraneoplastic MND. Males and females have similar distributions of the age at onset across their lifespan with no significant difference. Data are plotted in a scatter dot plot with mean. Males, *n* = 71, mean = 59.97 years, range = 29–79 years; Females, *n* = 61, mean = 59.34 years, range = 14–84 years. Two-tailed *t* test, *P* = 0.7750. The size of the sample (*n*) is the number of males or females for whom the age at onset is known. For 11 males and 7 females the age at onset is not reported. (**C**) Site of onset of paraneoplastic MND. The analysis shows the clinical site of onset, which is the bodily region where MND symptoms first present. For the site of onset, see Materials and Methods. The onset usually is focal or rarely multifocal. Of 163 patients, the site of onset is known for 105 patients. Lower limbs, *n* = 38; Upper limbs, *n* = 30; Bulbar, *n* = 12; Pseudobulbar, *n* = 1; Bulbar and pseudobulbar, *n* = 1; Upper and lower limbs, *n* = 8; Lower limbs and bulbar, *n* = 5; Upper limbs and bulbar, *n* = 2; Bulbar and cognitive, *n* = 1; Respiratory, *n* = 3; Multifocal (3 or more sites), *n* = 4. (**D**) MND phenotype of paraneoplastic MND. This is the MND phenotype as the disease evolves. ALS is the most common phenotype, followed by lower MND, whereas PLS is particularly rare. Of 163 patients, MND phenotype is known for 147 patients. ALS, *n* = 93; Lower MND, *n* = 49; PLS, *n* = 5. Data in A, C, and D are plotted in circle graphs. Each graph shows the relevant subcategories along with their percentage of occurrence, calculated as the number of patients displaying that feature to the total number of patients (shown at the bottom of graph) for whom there is available data for that feature.

**Figure 3:**
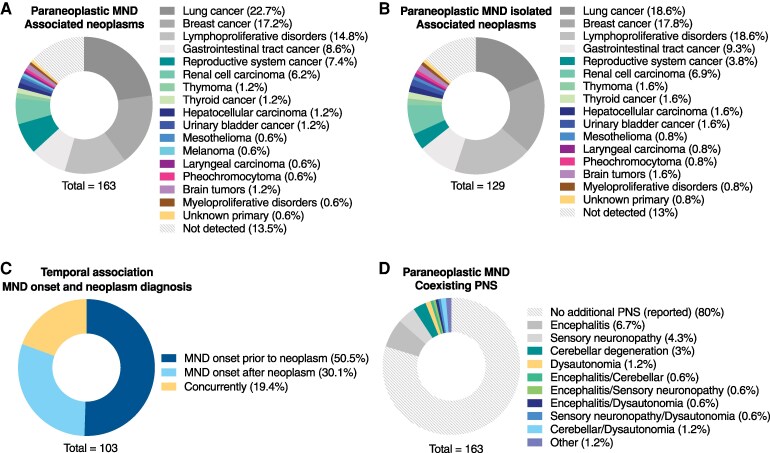
**Paraneoplastic MND is associated with various neoplasms and may present with other PNS.** (**A**) Neoplastic diseases encountered in the cohort of all paraneoplastic MND cases. A wide variety of solid tumors and hematologic malignancies are associated with MND. Of 163 patients, a neoplastic disease is detected in 141 patients. Lung cancer, *n* = 37; breast cancer, *n* = 28; lymphoproliferative disorders, *n* = 24; gastrointestinal tract cancer, *n* = 14; reproductive system cancer, *n* = 12; renal cell carcinoma, *n* = 10; thymoma, *n* = 2; thyroid cancer, *n* = 2; hepatocellular carcinoma, *n* = 2; urinary bladder cancer, *n* = 2; mesothelioma, *n* = 1; melanoma, *n* = 1; laryngeal carcinoma, *n* = 1; pheochromocytoma, *n* = 1; brain tumors, *n* = 2; myeloproliferative disorders, *n* = 1; unknown primary, *n* = 1; not detected, *n* = 22. (**B**) Neoplastic diseases encountered in the cohort of isolated paraneoplastic MND cases (in the absence of additional PNS). Of 129 patients, a neoplastic disease is detected in 112 patients. Lung cancer, *n* = 24; breast cancer, *n* = 23; lymphoproliferative disorders, *n* = 24; gastrointestinal tract cancer, *n* = 12; reproductive system cancer, *n* = 5; renal cell carcinoma, *n* = 9; thymoma, *n* = 2; thyroid cancer, *n* = 2; hepatocellular carcinoma, *n* = 2; urinary bladder cancer, *n* = 2; mesothelioma, *n* = 1; laryngeal carcinoma, *n* = 1; pheochromocytoma, *n* = 1; brain tumors, *n* = 2; myeloproliferative disorders, *n* = 1; unknown primary, *n* = 1; not detected, *n* = 17. (**C**) Temporal association of MND symptom onset with neoplastic disease diagnosis. Among all reviewed cases, this association is known for 103 patients. In most cases MND precedes neoplasm diagnosis (*n* = 52), less frequently it follows neoplasm diagnosis (*n* = 31) and to a lesser extent the two entities present concurrently (*n* = 20). (**D**) In 20% of cases (*n* = 33, of 163), MND presents with other PNS, which include encephalitis (*n* = 11), sensory neuronopathy (*n* = 7), cerebellar degeneration (*n* = 5), autonomic failure (*n* = 2) or combinations thereof (encephalitis/cerebellar, *n* = 1; encephalitis/sensory neuronopathy, *n* = 1; encephalitis/dysautonomia, *n* = 1; sensory neuronopathy/dysautonomia, *n* = 1; cerebellar/dysautonomia, *n* = 2). Two cases presented with other autoimmune conditions possibly paraneoplastic, including myasthenia (*n* = 1) and neuromyotonia (*n* = 1). Data are plotted in circle graphs. Each graph shows the relevant subcategories along with their percentage of occurrence, calculated as the number of patients displaying that feature to the total number of patients (shown at the bottom of graph) for whom there is available data for that feature.

**Figure 4:**
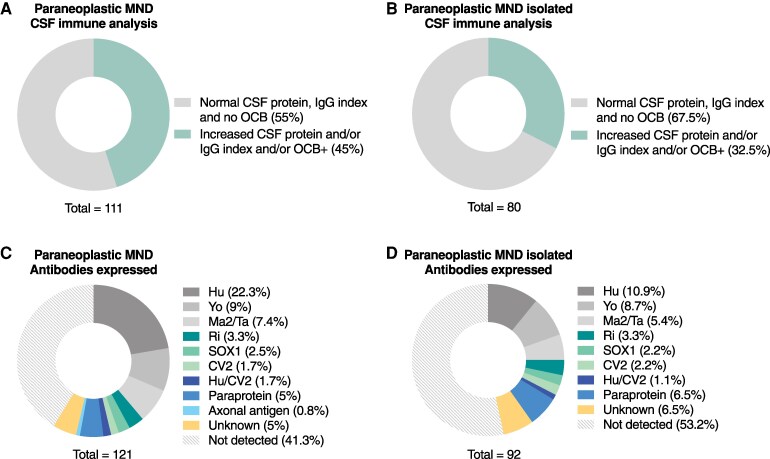
**Immune profiling of paraneoplastic MND in serum and CSF.** (**A**) Evaluation of immune markers in CSF, including total protein, IgG index and/or oligoclonal bands (OCB), in the cohort of all paraneoplastic MND cases. Of 111 patients for whom data is available, 45% (*n* = 50) display signs of inflammation in CSF, including elevated protein and/or elevated IgG index and/or CSF-restricted oligoclonal band expression, while 55% (*n* = 61) display CSF inflammatory markers within the reference range. (**B**) Evaluation of immune markers in CSF, including total protein, IgG index and/or oligoclonal bands, in the cohort of isolated paraneoplastic MND cases. Of 80 patients for whom data is available, 32.5% (*n* = 26) display immune derangements in CSF, including elevated protein and/or elevated IgG index and/or CSF-restricted oligoclonal band expression, while 67.5% (*n* = 54) have no CSF immune perturbations. (**C**) Antibodies expressed in the cohort of all paraneoplastic MND cases. Of 121 patients for whom antibody testing is reported, including onconeural antibodies or paraproteins in the case of lymphoproliferative disorders, 58.7% (*n* = 71) express an antibody. A variety of antibodies, most of which onconeural, are expressed in serum and/or CSF of these patients. Hu, *n* = 27; Yo, *n* = 11; Ma2/Ta, *n* = 9; Ri, *n* = 4; SOX1, *n* = 3; CV2, *n* = 2; Hu/CV2, *n* = 2; paraprotein, *n* = 6; axonal antigen, *n* = 1; not specified by authors, *n* = 6. Onconeural antibody was not detected in *n* = 50 patients. Note that in one case serum immunoreactivity is directed against novel neural antigens expressed in the neuron’s axon. (**D**) Antibodies expressed in the cohort of isolated paraneoplastic MND cases. Of 92 patients for whom antibody testing is reported, including onconeural antibodies or paraproteins, 46.8% (*n* = 43) express an antibody. Hu, *n* = 10; Yo, *n* = 8; Ma2/Ta, *n* = 5; Ri, *n* = 3; SOX1, *n* = 2; CV2, *n* = 2; Hu/CV2, *n* = 1; paraprotein, *n* = 6; not specified by authors, *n* = 6. Onconeural antibody was not detected in *n* = 49 patients. Data in A–D are plotted in circle graphs. Each graph shows the relevant subcategories along with their percentage of occurrence, calculated as the number of patients displaying that feature to the total number of patients (shown at the bottom of graph) for whom there is available data for that feature.

**Figure 5:**
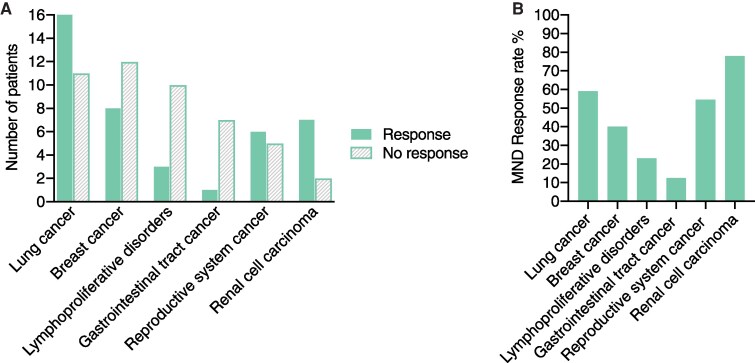
**Response of MND to antineoplastic and immune treatments.** (**A**, **B**) Response of MND associated with different neoplastic diseases to antineoplastic and/or immune treatments. Response is presented for MND associated with the six neoplastic diseases most commonly encountered in association with MND. (**A**) Quantification of neurologic response, presented as number of patients who respond or do not respond to the treatment of malignant disease and/or immune treatments as an absolute value, for different neoplastic diseases. Data are plotted in columns. Lung cancer, response *n* = 16, no response *n* = 11, NA, *n* = 10; Breast cancer, response *n* = 8, no response *n* = 12, NA, *n* = 8; Lymphoproliferative disorders, response *n* = 3, no response *n* = 10, NA *n* = 11; Gastrointestinal tract cancer, response *n* = 1, no response *n* = 7, NA *n* = 6; Reproductive system cancer, response *n* = 6, no response *n* = 5, NA *n* = 1; Renal cell carcinoma, response *n* = 7, no response *n* = 2, NA *n* = 1. NA stands for not available, which means no available data on treatment or treatment not received; these are not included in the analysis and the graph. (**B**) Quantification of neurologic response, presented as response rate %, calculated as the ratio of the number of responders divided by the sum of responders and non-responders ×100. This percentage is calculated separately for different neoplastic diseases and data are plotted in columns. The tumors with the highest response rates are renal cell carcinoma (78%), lung cancer (59%) and reproductive system cancer (54.5%), followed by breast cancer (40%), lymphoproliferative disorders (23%) and gastrointestinal tract cancer (12.5%). The neoplasms are presented along the *x*-axis with a decreasing frequency of their occurrence in association with MND. Response is considered the persistent, transient or partial neurologic response or stabilization of neurologic symptoms.

To explore the clinical and laboratory profiles of paraneoplastic MND, we pursued a systematic review of the literature on all relevant cases published to date. The retrieved cases were screened based on PNS-Care criteria,^4^ with modifications adopted for MND, and with application of inclusion and exclusion criteria, as outlined in Materials and Methods. The PNS-Care criteria adopted for MND allowed us to formulate the paraneoplastic ALS (pALS) score for paraneoplastic MND. The pALS score was calculated for each case and cases of all degrees of diagnostic certainty (definite, probable and possible) are included. The workflow of study screening and selection is reported according to the PRISMA guidelines (Fig. 1).^12^ For each individual, select disease items (or characteristics) are collected based on data availability (Supplementary Table 1). Descriptive statistics is used to perform meta-analysis of disease features on an individual patient basis, and data are presented in Figs 2–5.

### Demographic and clinical features of paraneoplastic MND

In our reviewed series of patients with paraneoplastic MND, 55% are males and 45% are females (Fig. 2A). The mean age at onset is ∼60 and 59 years for males and females, respectively (Fig. 2B). Analysis of the clinical site of MND onset revealed that the most common site of onset is lower limbs, followed by upper limbs, medulla, upper and lower limbs concomitantly, and lower limbs with bulbar symptoms concomitantly, with other patterns of onset being observed at lower degrees (Fig. 2C). These data are similar to the site of onset of idiopathic MND (Koropouli *et al*., unpublished).^45^ Next, we analyzed the MND phenotype. It has been postulated that in MND associated with lymphoproliferative disorders lower motor neurons are selectively involved (spinal and/or bulbar MND).^46,47^ However, subsequent studies have reported various MND phenotypes associated with lymphoproliferative disorders,^26,48-52^ or solid tumors and onconeural antibodies.^8,53-57^ There also seems to be no association between lymphoproliferative disease subtype and MND phenotype.^48,51^ This analysis in our reviewed series of paraneoplastic MND patients shows that 63.3% have ALS, 33.3% have lower MND, and 3.4% have PLS (Fig. 2D). In a few cases lower MND may present with a focal phenotype, including isolated vocal cord paresis, brachial paresis or diaphragmatic weakness.^58-63^ Further, isolated analysis for lymphoproliferative diseases and lung cancer demonstrated no particular predilection of these neoplastic diseases for select MND phenotypes (data not shown). Therefore, paraneoplastic MND spans the whole disease spectrum, in agreement with other studies reporting no consistent phenotypic difference between paraneoplastic and idiopathic MND.^9,34,64^ Taken together, the site of MND onset and the MND phenotype cannot serve as clinical markers for the paraneoplastic nature of MND.

### Neoplastic diseases and PNS associated with MND

We next asked which neoplastic diseases are associated with MND. Paraneoplastic MND may present along with a variety of neoplastic diseases that include solid tumors, lymphoproliferative or myeloproliferative disorders (Fig. 3A). Among these, lung cancer, breast cancer and lymphoproliferative disorders are most commonly encountered, followed with a decreasing frequency by gastrointestinal tract cancer, reproductive system cancer and renal cell carcinoma. Thymoma,^65^ thyroid carcinoma, hepatocellular cancer, urinary bladder cancer, mesothelioma, melanoma, laryngeal carcinoma, pheochromocytoma, myeloproliferative disorders,^66^ and unknown primary are detected at even lower frequencies. Primary brain tumors have been associated with MND in two patients.^67,68^ In 13.5% of cases there is no malignant disease detected up to the last follow-up. The same analysis for the cohort of isolated paraneoplastic MND cases, which are MND cases with no additional PNS, yielded similar results (Fig. 3B). To address the temporal relation of MND symptom onset with cancer diagnosis, we quantified the three different possibilities, and found that MND symptoms preceded neoplasm diagnosis in 50.5%, followed neoplasm diagnosis in 30.1% and presented concurrently with the neoplasm in 19.4% (Fig. 3C).

Not infrequently, patients with paraneoplastic MND present with involvement of additional neural sites resulting in a constellation of different PNS.^37^ Therefore, we sought to explore the PNS that present along with MND. In our reviewed series of patients, 18.8% of patients presented concomitantly with MND and another PNS, which most often included encephalitis, sensory neuronopathy, cerebellar degeneration and dysautonomia, while combinations thereof presented to lesser extents (Fig. 3D).

### CSF analysis and onconeural antibodies in paraneoplastic MND

We next asked whether there is immune dysregulation in paraneoplastic MND. To address this, we first looked for inflammation in CSF of paraneoplastic MND-affected individuals. Among paraneoplastic MND subjects for whom CSF analysis is reported (at least cell count and protein), 45% displayed CSF immune derangements including protein exceeding reference levels and/or elevated IgG index and/or CSF-specific oligoclonal bands (OCB) (Fig. 4A), whereas 32.5% displayed elevated markers of inflammation in the cohort of isolated paraneoplastic MND (Fig. 4B). Further, we analyzed onconeural antibody expression as a marker of immune activation. This analysis showed that of the subjects tested, 58.7% harbored antibody expression in serum and/or CSF among all paraneoplastic MND cases, whereas 46.8% harbored onconeural antibodies among isolated paraneoplastic MND cases. Of note, there is lower incidence of antibody expression among isolated paraneoplastic MND cases, compared with all paraneoplastic MND cases that include those with additional PNS. The most commonly expressed antibodies are those directed against Hu, Yo, Ma2, followed by Ri, SOX1 and CV2, for both the cohort of paraneoplastic MND (Fig. 4C) and the cohort of isolated paraneoplastic MND (Fig. 4D). Exceptionally, two onconeural antibodies may be expressed.^69^ Our data are consistent with data derived from large PNS series.^70^ Of note, two of the three SOX1-positive paraneoplastic MND cases are originally reported in this study with the first one reported elsewhere.^71^ When MND coexists with another PNS, it is unclear whether onconeural antibody expression is related to paraneoplastic MND and/or the co-extant PNS. This is partially elucidated by cases where an onconeural antibody expressed at high titers upon the onset of the first PNS, decreases to undetectable levels upon treatment, but it increases again upon the manifestation of a second PNS, even in the absence of neoplasm recurrence or of a new neoplasm.^37^ Of note, patients tested negative for known onconeural antibodies are not necessarily devoid of immunoreactivity against other, yet unidentified, neural antigenic epitopes, as exemplified elsewhere.^72,73^ Immunoreactivity against acetylcholine receptor is of unclear significance.^74^

### Diagnosis and treatment of paraneoplastic MND

Paraneoplastic MND diagnosis is made based on diagnostic criteria for PNS^4,10^ and requires a high degree of clinical suspicion.^15,64^ The core criterion is considered the diagnosis of MND and malignant disease with a close temporal association (2 years or less) along with neurologic response to antineoplastic treatment.^32^ The latter provides proof of concept for the paraneoplastic origin of MND. In the absence of antineoplastic therapy-induced neurologic remission, an MND course parallel to the course of neoplastic disease, the concurrent presence of another PNS and onconeural antibody expression strongly support paraneoplastic MND diagnosis.^64,75-77^ Paraneoplastic MND has a rapidly progressive course,^5,8,78-83^ instead of the chronic course of idiopathic MND, which is of diagnostic value. Despite that onconeural antibodies are robust PNS biomarkers, only a subset of patients expresses onconeural antibodies in serum and/or CSF (Fig. 4).^84^ As such, their absence does not rule out paraneoplastic MND diagnosis^9,85,86^ and it should prompt efforts for the discovery of novel immune reactivity, as reported elsewhere.^72,73^ CSF analysis is important for evaluating for neoplastic dissemination into the CNS and for CNS inflammation including increased white cell count, protein, albumin index, IgG index and expression of CSF-specific oligoclonal bands or onconeural antibodies. With all these considerations, we formulate diagnostic recommendations for paraneoplastic MND (Table 1). Upon PNS consideration, a rigorous work-up should be conducted for underlying neoplastic diseases,^87^ including CT imaging of the chest and abdomen, testing for monoclonal immunoglobulin expression, bone marrow biopsy,^50,51,88,89^ gastrointestinal tract endoscopy, mammography, and testicular ultrasound especially in the case of anti-Ma2 antibody expression.^55^ In case of negative work-up,^90,91^ whole-body 18-FDG-PET-CT can significantly increase the diagnostic yield for neoplasms.^24^ If PET-CT is unrevealing, screening with the above methods every 6 months and annual assessments with PET-CT scans are advocated.

**Table 1:** Diagnostic recommendations for paraneoplastic MND

Paraneoplastic MND diagnostic recommendations
Co-occurrence of MND and neoplastic disease
MND course associated with tumor progression or recurrence
Fulminant or rapidly progressive MND
Onconeural antibody expression
Co-occurrence of another paraneoplastic neurologic syndrome
Neurologic response to cancer treatment and/or immune therapy

A qualitative diagnostic guide for paraneoplastic MND is formulated based on data derived from case review and meta-analysis combined with consensus PNS diagnostic criteria. These considerations allow the development of a combinatorial diagnostic approach based on clinical and laboratory markers. The close temporal association of MND and cancer may be regarded as the core criterion for paraneoplastic MND diagnosis, whereas neurologic response to antineoplastic therapy provides proof of concept. Provided that PNS and cancer may present several years apart and often PNS don’t respond to antineoplastic treatment, the core and proof of concept criteria cannot be used to definitively rule out paraneoplastic MND diagnosis, especially in the presence of strong additional factors that include onconeural antibody expression and/or co-extant PNS. Exquisitely rapid progression is the only distinctive clinical feature of paraneoplastic MND, and may raise the question of this syndrome in the absence of additional supportive criteria.

Prompt initiation of antineoplastic therapy and immune therapy constitute the mainstay of PNS treatment. Immune therapy includes short-term immunotherapy that may be intravenous steroids, plasma exchange (PLEX) or IVIG, and long-term immunotherapy.^92^ PNS have a grave prognosis and in most instances they are unresponsive to any treatment,^93-96^ but instead they follow a progressive course.^29,97,98^ To examine this in our reviewed series of MND patients, we quantified the response of MND to antineoplastic and/or immune treatments for the neoplastic diseases most commonly encountered in association with MND. This analysis revealed that renal cell carcinoma, lung cancer and reproductive system cancer are associated with the highest rates of neurologic response, whereas tumors of the gastrointestinal tract, lymphoproliferative disorders and breast cancer are associated with lower response rates (Fig. 5). Interestingly, renal cell carcinoma may exhibit sustained remission following tumor eradication,^14,20,99-102^ while similar responses have also been described for other neoplasms,^7,8,41,68,76,79,103-106^ in consistency with previous observations that PNS prognosis is dependent upon the type of malignancy and onconeural antibody.^55,107^ Paraneoplastic MND is a major factor of morbidity and mortality since a lot of these patients survive cancer but die of MND.^9,48,93^

### Paraneoplastic MND sheds light on MND pathophysiology and reveals a continuum in the MND spectrum

**Figure 6:**
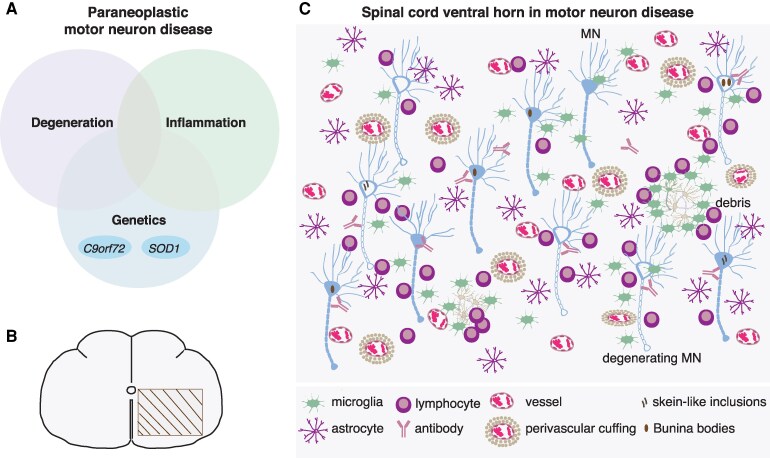
**Pathophysiology of paraneoplastic MND.** (**A**) Paraneoplastic MND emerges from the crosstalk of inflammatory, degenerative and genetic pathways. These pathways converge and lead to severe motor neuron dysfunction and neuronal death. The *C9orf72* and *SOD1* genetic loci, which are causally associated with MND, have been implicated in cases of MND associated with neoplastic diseases (Supplementary Table 1). (**B**, **C**) Pathological perturbations in motor neuron (MN) pools in paraneoplastic or idiopathic MND. In C, schematic representation of the pathological processes that take place in spinal cord ventral horns (rectangular selection in B) or motor nuclei of medulla, in MND, as these have been evidenced by autopsy studies of affected individuals (Supplementary Table 1). Inflammatory and degenerative processes evolve at the same time, and contribute to neuronal death in a synergistic manner. An immune trigger induced by malignant disease activates both intracellular and extracellular non-cell autonomous processes. Motor neuron degeneration further activates the immune system to remove cellular debris, which combined with a low capacity of motor neurons to withstand excitotoxic insults, establishes a vicious circle of inflammation and degeneration within motor neuron pools. Lymphocytes, antibodies, activated microglia and reactive astrocytes are the main inflammatory mediators in the microenvironment of motor neurons in MND. Lymphocytes may be clustered perivascularly forming a ‘perivascular cuff’ or may be accumulated around neurons. These histological alterations that have been described in paraneoplastic MND, have also been detected in idiopathic MND, which reveals a pathological continuum in MND spectrum disorders, in consistency with the similarities in the natural history of paraneoplastic and idiopathic variants of the disease. Of note, they have been described familial clustering or MND causative gene mutations in paraneoplastic MND, which further reveals the tight link between genetic susceptibility and extrinsic cues for the emergence and perpetuation of this complex disease. Abbreviations: *C9orf72*, chromosome 9 open reading frame 72; *SOD1*, superoxide dismutase 1; MN, motor neuron.

Select case studies report the manifestation of familial MND or MND harboring genetic deficits in *C9orf72* or *SOD1* genes upon the occurrence of neoplastic disease.^17,80,108,109^ These studies put forward the notion that inflammation is capable of inducing MND in genetically predisposed individuals, and reveal the critical role of inflammation in MND pathogenesis. These observations are consistent with isolated reports of immune-mediated MND.^43,105,110^ Neuropathological examination has been valuable for investigating paraneoplastic MND. The most prominent pathologic feature is marked loss of motor neurons in the brainstem and the ventral horns of the spinal cord.^28,31,47,48,111-113^ On several but not all occasions, there is inflammation consisting primarily of T and B lymphocytes,^39,46,111,114^ which may surround blood vessels forming a perivascular ring known as ‘perivascular cuffing’.^115,116^ Of note, inflammatory and degenerative perturbations display a gradient starting from the site of onset and mitigated at a growing distance from the site of onset,^39,114^ with inflammatory cells being most abundant within CNS regions where motor neuron loss is most prominent and related with MND symptoms. The latter provides strong, yet indirect, evidence that inflammation contributes to or accompanies motor neuron degeneration. Nevertheless, these histological alterations and cytoplasmic inclusions of phosphorylated TDP-43^111^ and Bunina bodies that have been observed in paraneoplastic MND,^47,117^ (Fig. 6) are also histological hallmarks of idiopathic MND.^1,118^ Not infrequently, in paraneoplastic MND pathological changes extend beyond motor neuron-residing areas and affect dorsal root ganglia/dorsal columns^119^ or the cerebellum,^120^ even in the absence of clinically evident ataxia, which is usually not observed in idiopathic MND.^13,39,56,121^ Notably, the paraneoplastic immune process may follow an independent course, which is partly supported by remaining high titers of autoantibodies in spite of sustained tumor remission.^37^ These observations are accordant with studies showing activation of select inflammatory pathways in idiopathic ALS.^122-124^ The common pathological perturbations in idiopathic and paraneoplastic MND reveal that MND constitutes a continuous spectrum of MND disorders underlined by the convergence of degenerative and inflammatory mechanisms that gives rise to distinct pathological-clinical entities.^1,125^

## Discussion

This study provides strong evidence for the existence of MND of paraneoplastic origin. First, the four cases reported here expand the clinical and serological spectrum of paraneoplastic MND. Second, by means of a systematic review of all paraneoplastic MND cases with individual patient data meta-analysis of disease features, our work provides quantified data on various disease parameters, which can guide clinical practice, and simplifies clinical thought related to this rare PNS. Furthermore, our work elaborates on MND neuropathology and elucidates aspects of MND pathogenesis in paraneoplastic and non-paraneoplastic settings. With the emergence of an increasing number of case studies with paraneoplastic MND, there is growing recognition that MND is a PNS not dissimilar to other classical PNS. It shares the same clinical course, onconeural antibodies and immune dysregulation in CSF with other PNS. Paraneoplastic MND also shares several features with idiopathic MND at clinical, laboratory and pathological levels, which highlights that paraneoplastic MND constitutes part of MND spectrum disorders. The prevailing mechanism is apparently the convergence of neuroinflammatory, neurodegenerative and genetic pathways. Our work has certain limitations the most important of which is the retrospective analysis limited by the availability of data on certain disease features and for a subset of patients. Despite its limitations, our systematic analysis may aid in reshaping our clinical approach in diagnosing this PNS and may also contribute to the understanding of the pathogenesis of select MND cases in the landscape of an otherwise untreatable and lethal disease.

## Supplementary Material

fcag024_Supplementary_Data

## Data Availability

All raw data collected for the study, including patient-level data and a data dictionary defining each field in the set, are presented in Supplementary Table 1. There are no additional documents to be made available. These data will be available with publication of the article and constitute an integral part of the study.
