# Supplementary material for: Motor neuron disease can present as a paraneoplastic neurologic syndrome with various phenotypes
Source: Brain Commun. 2026 Mar 9;8(2):fcag024. doi: 10.1093/braincomms/fcag024 (PMC12967333; doi:10.1093/braincomms/fcag024)
Supplement: fcag024_Supplementary_Data [file fcag024_supplementary_data.pdf]

**Supplementary Table 1. Clinical and laboratory data of paraneoplastic motor neuron disease case studies**

| Case study | Age | Sex | Motor Neuron Disease Onset symptoms[a] MND phenotype[b] | Additional neurologic manifestations | CSF | Neoplasm (before MND, -x; after MND, +x) | Antibody | Neurologic response to antineoplastic or immune treatment |
|---|---|---|---|---|---|---|---|---|
| Henson et al, 1954 | 59 | F | Lower limb weakness[a]<br>ALS[b]<br>**Autopsy-proven:**<br>MN loss and inflammatory infiltrates in cervical and lumbar ventral horns, perivascular cuffing in spinal cord ventral horns/ subthalamic nucleus and cerebellum, degeneration of superior cerebellar peduncles. | Cerebellar syndrome, cranial nerve palsies | ↑protein | Breast cancer (with lymph node metastases) | | Progressive course. Died nine months after symptom onset. |
| | 55 | F | Bulbar, Lower limbs[a]<br>ALS[b]<br>**Autopsy-proven:**<br>MN loss and inflammatory infiltrates in cervical and lumbar ventral horns, perivascular cuffing in brainstem, degeneration and inflammation in pyramidal tracts, degeneration of spinal cord ventral roots and of cranial nerves III, IX, X, XII. | | Normal (cells, protein) | SCLC | | Progressive course. Died 20 months after MND symptom onset. |
| Rowland and Schneck, 1963 | 27 | F | Left lower limb weakness[a]<br>Lower MND[b]<br>**Autopsy-proven:**<br>Asymmetric MN loss in left ventral horns in cervical and lumbar spinal cord, astrocytosis, dorsal column degeneration. | | Normal | Hodgkin's lymphoma (+ 3 years) | | Died four years after MND onset |
| | 14 | F | Left upper limb weakness[a]<br>Lower MND[b]<br>**Autopsy-proven:**<br>MN loss in cervical/thoracic/ lumbar spinal cord ventral horns, astrocytosis, microglial reaction, perivascular lymphocytes, dorsal column degeneration. | | Normal | Hodgkin's lymphoma (at autopsy) | | Died 14 months after MND onset |
| Brain et al, 1965 | 78 | M | Lower limbs[a]<br>Lower MND[b] | | | Lung cancer (concurrently) | | Progressive course. Died seven months after MND symptom onset. |
| | 53 | M | Lower limbs[a]<br>ALS[b]<br>**Autopsy-proven:**<br>Gliosis and lower cell density in spinal cord ventral horns, CST and dorsal column/DRG degeneration. | Cerebellar ataxia | ↑protein | Lung adenocarcinoma (concurrently) | | Response to tumor excision. Died of cancer progression 21 months after MND symptom onset. |
| Walton et al, 1968 | 44 | M | Left upper limb[a]<br>Lower MND[b]<br>**Autopsy-proven:**<br>MN loss, perivascular inflammation, lymphocytosis and microglia in spinal cord ventral horns. | | | Hodgkin's lymphoma (-2 years) | | Progressive course. Died three months after MND symptom onset. |

| | | | | | | | |
|---|---|---|---|---|---|---|---|
| Adams et al, 1970 | 56 | F | Upper limb and neck weakness[a]<br>Lower MND[b]<br>**Autopsy-proven:**<br>Cervical spinal cord: ventral and dorsal horn cell loss, degenerated anterior roots, perivascular lymphocytic infiltrates, astrocytic hyperplasia. Hypoglossal nucleus cell loss, degeneration of acoustic nerve and cochlear nuclei. | Sensory disturbances | Normal | SCLC | Progressive course |
| Buchanan and Malamud, 1973 | 36 | M | Right lower limb[a]<br>ALS[b]<br>**Autopsy-proven:**<br>MN loss and reactive gliosis in spinal cord ventral horns (most severe on right side of lumbar cord), MN loss in hypoglossal nucleus bilaterally, demyelination of right lumbar ventral roots. | | Normal | Renal cell carcinoma (+32 months) | Response to cancer treatment. Died of metastatic renal cancer seven years after cancer diagnosis. |
| Smith et al, 1975 | 36 | M | Limb weakness[a]<br>ALS[b]<br>**Autopsy-proven:**<br>CST degeneration most prominent in cervical and thoracic spinal cord, MN loss and gliosis in spinal cord ventral horns and in hypoglossal nuclei, astrocytosis and Rosenthal fibers in CST and spinal cord ventral horns, spinal cord and ventral root thinning. | | | Glioblastoma multiforme<br>(right frontal lobe) | Died of glioblastoma multiforme |
| Bauer et al, 1977 | 77 | M | Left lower limb[a]<br>ALS[b]<br>**Autopsy-proven:**<br>Spinal cord ventral horn MN loss and gliosis, ventral root thinning, degeneration of ventral and lateral tracts, sparing of dorsal roots, muscle fiber atrophy. | | Normal | Waldenström macroglobulinemia (probably macroglobulinemia preceded MND) | Progressive course. Died of respiratory failure two years after MND onset. |
| Peacock et al, 1979 | 64 | M | Upper limbs[a]<br>ALS[b] | | | Squamous cell lung carcinoma | Response to cancer treatment (radiation therapy) |
| Mitchell and Olczak, 1979 | 54 | M | Lower limbs[a]<br>ALS[b] | | Normal | Large cell lung cancer | Strong response to cancer treatment |
| Gritzman et al, 1983 | 69 | M | Bulbar[a]<br>ALS[b]<br>**Autopsy-proven:**<br>MN loss in spinal cord ventral horns and hypoglossal nuclei, loss of axons in ventral roots, frontal lobe perivascular inflammation and reactive | | | Esophageal carcinoma (squamous, metastatic)<br>(+27 months) | Fulminant course. Died 27 months after symptom onset. |

| | | | | | | | |
|---|---|---|---|---|---|---|---|
| | | | microglia. | | | | |
| | 74 | M | Right upper limb[a]<br>ALS[b] | Normal | Rectal adenocarcinoma (concurrently) | | |
| Thomas et al, 1984 | 53 | M | Orthopnea[a]<br>Diaphragmatic paralysis (bilateral),upper limb weakness[b]<br>**Autopsy-proven:**<br>Phrenic nerve axonal ballooning, sparse perivascular lymhocytic infiltrate in cervical spinal cord, neurogenic atrophy of diaphragm | | Renal cell carcinoma (at autopsy) | | Died 19 months after symptom onset (inferior vena cava thrombosis) |
| Recine et al, 1984 | 54 | F | Lower limb weakness[a]<br>Lower MND (spinal)[b]<br>**Autopsy-proven:**<br>MN loss in ventral horns, mild cell loss in dorsal horns, segmented demyelination in dorsal roots/columns, increased Schwann cells in roots. | Patient denied lumbar puncture | Hodgkin's disease (-3 months) | | Fulminant course. Died five months after lymphoma diagnosis and two months after MND symptom onset. |
| Stoll et al, 1984 | 51 | M | Limb weakness[a]<br>Lower MND[b] | Normal | Thymoma (recurrence) | Not detected | No response to tumor treatment (radiation) |
| Evans et al, 1990 | 74 | M | Lower limb weakness[a]<br>ALS[b] | Normal cell count, protein | Renal cell carcinoma (+7 months) | | Response to nephrectomy (two-year follow-up) |
| Hays et al, 1990 | 73 | F | Lower limbs[a]<br>ALS (predominantly lower limbs)[b]<br>**Autopsy-proven:**<br>MN loss and astrocytosis in ventral horns (mostly in lumbar spinal cord), ventral root thinning, Bunina bodies, pallor and astrocytosis in lateral CST. IgA immune reactivity in lumbar ventral horns, IgA binding to axons and cell bodies in CNS and PNS. IgA bound to neurofilaments in vitro. | Normal<br>OCB (-) | Breast cancer<br>(-2 years)<br>(metastatic at autopsy) | Monoclonal IgA, anti-GM1 (low titer) | Progressive course.<br>Died of cardiac arrhythmia. |
| Younger et al, 1991 | 63 | M | ALS[b] | Normal | Chronic lymphocytic leukemia | IgM-κ | No treatment. Progressive course. Died three years later (quadriparesis, aspiration). |
| | 61 | M | ALS[b]<br>**Autopsy-proven:**<br>MN loss in spinal cord ventral horns, brainstem and motor cortex, bilateral CST degeneration. Axonal degeneration and inflammatory infiltrates in peripheral nerves. | ↑ Protein<br>OCB (+) | Hodgkin's disease (at autopsy) | No paraprotein | Progressive course. Died three years later (quadriparesis, aspiration, sepsis) |
| | 73 | F | ALS[b] | Normal | Chronic lymphocytic leukemia | No paraprotein | No treatment. Progressive |

| | | | | | | | | |
|---|---|---|---|---|---|---|---|---|
| | | | | | | (-2 years) | | course. |
| Dalmau et al, 1992 | 69 | M | Head drop, gait disorder[a]<br>ALS[b] | Encephalitis (seizures, nystagmus, diplopia) | ↑ Protein<br>OCB (+) | SCLC | Anti-Hu (serum) | Died of respiratory failure |
| | 60 | F | Bulbar, limb weakness, respiratory muscle weakness[a]<br>Lower MND[b]<br>**Autopsy-proven:**<br>MN loss, perivascular and interstitial inflammatory infiltrates and microglial nodules in spinal cord ventral horns, medulla and amygdala (highest content of anti-Hu antibody). | Cerebellar syndrome | | SCLC | Anti-Hu (serum and CSF) | |
| | 70 | M | Lower limb weakness[a]<br>Lower MND[b]<br>**Autopsy-proven:**<br>MN loss in ventral horns, DRG neuron loss and dorsal column degeneration, inflammation and gliosis in spinal cord gray matter. | Sensory neuronopathy, cranial nerve palsy, dysautonomia | ↑ Protein | Prostate carcinoma (poorly differentiated) | Anti-Hu (serum and CSF) | Died of pulmonary embolism six months after neurologic symptom onset |
| Rowland et al, 1995 | 65 | M | Left upper limb weakness[a]<br>ALS[b]<br>*Rapidly progressive*<br>**Autopsy-proven:**<br>MN loss and astrocytosis in ventral horns and motor cortex, CST degeneration, loss of large myelinated fibers in ventral roots. | | Normal | Waldenström's macroglobulinemia | Monoclonal IgM-κ, Anti-SGPG (high titer) | No response to PLEX or antineoplastic therapy. Died one year after MND symptom onset. |
| Verma et al, 1996 | 51 | M | Upper limb weakness[a]<br>ALS[b]<br>*Rapidly progressive*<br>**Autopsy-proven:**<br>MN loss in ventral horns (cervical>lumbar), IgG immunoreactivity in medullary nuclei, Purkinje cell loss. | | ↑ Cell count<br>↑ Protein | SCLC (pleural metastasis) (concurrently) | Anti-Hu (serum and CSF) | No response to cancer or immune treatment (corticosteroids ,IVIG,PLEX, cyclophosphamide). Died 23 months after MND symptom onset. |
| Forsyth et al, 1997 | 60 | F | PLS[a] | Urinary urgency | OCB (-) | Breast cancer (+1 month) | Not detected | Progressive course |
| | 45 | F | PLS (spastic dysarhtria)[a] | | ↑ Protein<br>OCB (-) | Breast cancer (+0.5 month) | Not detected | No response to steroids. Progressive course. |
| | 63 | F | PLS[a]<br>ALS[b] | | | Breast cancer (+3 months) | Not detected | Died of respiratory failure 24 months after MND onset. |
| | 70 | F | Bulbar[a]<br>ALS[b] | Dementia | Normal | Breast cancer (-6 months) | Not detected | No response to tumor treatment. Died 18 months after MND onset. |
| | 63 | M | Bulbar[a]<br>ALS[b] | | Normal | Hodgkin's lymphoma (+3 months) | Not detected | No response to tumor treatment |
| | 59 | F | Lower limbs[a] | | Normal | Uterine cancer | Not | No response to |

| | | | | | | | | |
|---|---|---|---|---|---|---|---|---|
| | | | ALS[b] | | | (-24 months) | detected | tumor treatment, alive at 40-month follow-up. |
| | 73 | F | Lower limbs[a] ALS[b] | | Normal | Uterine cancer (-4 months) | Not detected | Subjective response to radiation. Died 27 months after MND onset. |
| Herrero et al, 1998 | 62 | F | Limb weakness[a] ALS[b] | | Normal | Mantle cell lymphoma (leukemic phase) | No paraprotein | No response to lymphoma treatment. |
| Khwaja et al, 1998 | 67 | F | Upper limb weakness[a] ALS[b] *Rapidly progressive* | | Normal | Ovarian adenocarcinoma with peritoneal metastases (concurrently) | Anti-Yo (PCA-1) (serum) | No response to tumor debulking, progressive course. |
| Correa et al, 1999 | | | MND[b] | Sweet's syndrome | | Esophageal epidermoid carcinoma (+x) | | |
| Forman et al, 1999 | 70 | F | Hypercapnic respiratory failure[a] Lower MND[b] | | Normal (cells, protein) | Renal cell carcinoma (granular) | Negative anti-Hu, no paraprotein | Complete response to cancer excision (one-year follow-up) |
| Ferracci et al, 1999 | 72 | F | Left upper limb weakness[a] Lower MND (spinal and brainstem)[b] | Nystagmus, dizziness | ↑ Protein OCB (+) | Breast ductal adenocarcinoma with lymph node infiltration (+4 months) | Directed against axon initial segments and nodes of Ranvier (serum) | No response to immune therapy (IVIG, PLEX, i.v. methylprednisolone, azathioprine). Partial response to cancer resection. |
| Berghs et al, 2001 | | | | | | | Anti-spectrin | |
| Rijnders and Decramer, 2000 | 52 | M | Left diaphragmatic paralysis[a] Bilateral diaphragmatic paralysis[b] | | | Renal cell carcinoma | | Partial response to cancer treatment (improvement of respiratory function). |
| Vigliani et al, 2000 | 65.3 (mean, range 52-84) | M(9) F(5) | Spinal[a](10), Bulbar[a](4) ALS[b] | | ↑ Protein (1/4) | Breast cancer (4), lung adenocarcinoma (3), intestinal (3), mesothelioma (1), hepatocellular (1), renal (1), unknown primary metastatic (1) In 8: +11 months (mean) In 3: concurrently In 3: -5, -12, -19 months | Not detected | No response to cancer treatment. Eight patients died of MND-associated respiratory failure after a mean of 18 months. |
| Bir et al, 2000 | 64 | M | Upper limbs, bulbar[a] Lower MND[b] | | ↑ Protein | Myelofibrosis (concurrently) | | Improvement following hydroxyurea treatment. Stable at one-year follow-up). |

| | | | | | | | | |
|---|---|---|---|---|---|---|---|---|
| Viera-Alemán et al, 2002 | 60 | F | Left lower limb[a]<br>ALS[b]<br>*Rapidly progressive* | | | Renal cell carcinoma (+10 months) | | No response to cancer treatment. |
| Khealani et al, 2004 | 57 | M | Lower limb weakness[a]<br>Lower MND[b] | Sensory ataxia | | Esophageal large cell adenocarcinoma (concurrently) | | Conservative treatment. Died of pneumonia two months after presentation. |
| Ogawa et al, 2004 | 80 | F | Muscle weakness and atrophy[a]<br>Upper/lower MND[b]<br>(Upper MND in autopsy only)<br>**Autopsy-proven:**<br>Loss of Betz cells, pyramidal tract degeneration bilaterally with macrophages, cell loss in ventral horns/DRG/ brainstem/cerebellum, dorsal column degeneration with macrophages, reactive astrocytosis, scarce perivascular lymphocytic infiltration. | Sensory neuronopathy<br>Facial nerve palsy | ↑ Cell count<br>↑ Protein<br>OCB (-) | Gall bladder adenocarcinoma<br>Duodenum small cell carcinoma | Anti-Hu | Died of respiratory failure eight months after neurological symptom onset |
| Jeon et al, 2004 | 55 | M | Limb weakness and atrophy[a]<br>Lower MND (spinal, bulbar)[b] | | ↑ Protein<br>↑ IgG index | SCLC<br>(+6 months) | Not detected | Rapidly progressive course |
| Chang et al, 2004 | 49 | F | Bulbar[a]<br>ALS (bulbar)[b]<br>Bilateral vocal cord paresis | Encephalitis | Normal | SCLC | Anti-Hu | Response to cancer treatment (chemotherapy) , Improvement of vocal cord function. |
| Kijima et al, 2005 | 52 | F | Upper limb weakness[a]<br>MND[b]<br>*Progressive MND until cancer diagnosis and treatment* | | | Invasive ductal breast cancer HER2-positive (+6 years) | | Stabilization after cancer treatment |
| Gazic et al, 2005 | 68 | F | Limb weakness[a]<br>Lower MND[b]<br>*Rapidly progressive*<br>**Autopsy-proven:**<br>Spinal cord ventral horn: MN loss (cervical/thoracic> lumbar), perivascular CD8+ T lymphocytes surrounding MNs, mononuclear clusters, glial proliferation,macrophages.<br>Dorsal root ganglia/columns: DRG neuron loss, Nageotte's nodules, mononuclear clusters, CD8+ T cells surrounding sensory neurons, abundant CD68+ macrophages in DRG, perivascular lymphocytic infiltrate in dorsal columns, dorsal column degeneration.<br>Brain: neuronophagia, microglial nodules, perivascular cuffing, perineuronal CD8+ T lymphocytes. | Encephalitis, sensory neuronopathy, facial nerve palsy | ↑ Protein | SCLC | Anti-Hu (serum and CSF) | Fulminant course, respiratory failure/mechanical ventilation. No response to IVIG. Died six months after symptom onset. |
| Hays et al, 2006 | 66 | F | Lower limbs[a]<br>ALS[b] | | Normal | Breast cancer<br>(-12 months) | Not detected | No response to IVIG, PLEX or |

| | | | | | | | | |
|---|---|---|---|---|---|---|---|---|
| | | | *SOD1 I113T*<br>**Autopsy-proven:**<br>Ventral horn and root atrophy, severe MN loss in ventral horns and hypoglossal nuclei, Betz cell loss in precentral gyrus, pyramidal tract pallor, scarce macrophages in thoracic spinal cord, conglomerate hyaline cytoplasmic inclusions (strong neurofilament and peripherin and slight ubiquitin staining), dorsal column pallor. | | | | | cancer treatment. Died 14 months after MND symptom onset. |
| Waragai et al, 2006 | 36 | M | Head drop/Upper limbs[a]<br>MND[b] | Encephalitis (amnesia, seizures, gaze palsy, irritability) | ↑ Protein | Testicular germ cell tumor (-14 months from MND,<br>-6 months from encephalitis) | Anti-Ma2 (serum and CSF) | Improvement with immune treatment (steroids, IVIG). Cancer was treated before PNS, no tumor recurrence. |
| Kararizou et al, 2007 | 57 | M | Lower limb weakness[a]<br>ALS[b] | | Normal | Prolactinoma | | Response to prolactinoma treatment (bromocriptine) |
| Sadot et al, 2007 | 51 | F | Bulbar[a]<br>ALS[b] | FTD | Normal | Breast adenocarcinoma (relapse) | Not detected | No response to cancer treatment, died of aspiration pneumonia 17 months after MND symptom onset |
| | 72 | F | Bulbar[a]<br>Upper-dominant ALS[b] | FTD | Normal | Breast adenocarcinoma (+1 month) | Not detected | |
| Turgut et al, 2007 | 66 | M | Right upper limb[a]<br>Lower MND (spinal, bulbar)[b] | | Normal | Hepatocellular carcinoma<br>(-2 months) | | Progressive course, died six months after MND symptom onset. |
| Sato et al, 2007 | 60 | M | Upper/lower limb weakness[a]<br>ALS[b] | | | Rectal cancer metastatic<br>(-x months, MND manifestation during adjuvant chemotherapy) | | Rapidly progressive course despite tumor response, died of aspiration pneumonia eight months after MND onset. |
| Cánovas et al, 2007 | | | Lower MND[a]<br>Lower MND[b] | Neuromyotonia | Not reported (abstract) | Clear renal cell carcinoma | | Response to tumor treatment |
| Martín et al, 2007 | 29 | M | ALS[a]<br>ALS[b] | | Not reported (abstract) | Not detected<br>(3-year follow-up) | Anti-CV2 | Not reported (abstract) |
| Hoffmann et al, 2008 | 70 | M | Lower limbs[a]<br>ALS[b] | | ↑ Protein | Not detected<br>(4-year follow-up) | Anti-Ta | Stable (four-year follow-up) |

| | | | | | | | | |
|---|---|---|---|---|---|---|---|---|
| Tofaris and Farmer, 2008 | 63 | F | Right upper limb[a]<br>Focal lower MND (C7-T1)[b]<br>No SCLC recurrence | | OCB+ | SCLC (treated)<br>(-20 months) | Anti-Hu | Stable, lower MND restricted to right upper limb |
| Koc and Yerdelen, 2008 | 70 | M | Right lower limb (foot drop)[a]<br>MND[b] | | Normal | Non-Hodgkin's lymphoma<br>(+12 months) | | Not reported |
| Turk et al, 2009 | 59 | M | Upper limbs[a]<br>MND[b]<br>In the case of both right and left kidney's cancer, weakness and fasciculations in upper limbs | | Not reported | Renal cell carcinoma (RCC) sequentially in both kidneys (first RCC: +5 months, second RCC: concurrently with MND relapse)<br>The two RCC presented 58 months apart. | | Complete response to tumor excision, in case of both right and left kidney's cancer |
| Distad and Weiss, 2010 | 54 | F | Limbs, bulbar[a]<br>Lower MND (spinal/bulbar)[b]<br>*Rapidly progressive* | Cerebellar syndrome | ↑IgG index<br>OCB(+) | Breast ductal carcinoma<br>(-1 month) | Anti-Yo | Patient denied treatment, progressive course |
| Ducray et al, 2010 | 47 | M | Upper limbs, trunk, abdomen[a]<br>Lower MND[b] | Sensory axonal polyneuropathy | ↑ Protein<br>OCB(+) | Undifferentiated bronchial carcinoma | Anti-Hu | Stabilization after treatment with IVIG, cyclophosphamide), stable eight years after MND onset. |
| | 42 | F | Lower limbs[a]<br>Lower MND[b] | Sensory neuronopathy | ↑ Protein | SCLC | Anti-Hu (increased upon MND onset) | No response to chemotherapy or to steroids, IVIG, cyclosporine. Died of respiratory failure five years later. |
| | 70 | M | Lower limbs[a]<br>Lower MND[b] | Brainstem encephalitis | ↑ Protein | Prostatic adenocarcinoma | Anti-Hu | Response to steroids, stable five years after MND onset. |
| | | | *In all three patients, MND presented as a second PNS, without evidence of cancer recurrence or of a new neoplasm* | | | | | |
| Briani et al, 2011 | 63, mean | M (2)<br>F (2) | MND[b] (3)<br>PLS[b] (1) | | Normal | Non-Hodgkin lymphoma (preceded by MND in 3 pt, followed by MND by 1 y in 1 pt) | Not detected | No response to lymphoma treatment (4/4) |
| Jurici et al., 2011 | 71 | F | Bulbar (dysarthria)[a]<br>ALS[b]<br>**Autopsy-proven:**<br>MN loss in ventral horns and hypoglossal nucleus,<br>CST atrophy, Bunina bodies in surviving MN, severe neuronal loss in frontal and temporal cortex. | | ↑ Protein | Waldenström macroglobulinemia | Anti-MAG IgM | No response to rituximab and chlorambucil. Progressive course. Died of aspiration pneumonia 20 months after symptom onset. |
| Piccolo et al., 2011 | 49 | F | Spastic paraparesis[a]<br>PLS[b] | Sjögren syndrome (anti-Ro+) | OCB(+) | Not detected (4-year follow-up)<br>(including with PET-CT until 1 year) | Anti-Ma2/Ta (serum and CSF) | Poor response to corticosteroids, PLEX. |

| | | | | | | | | |
|---|---|---|---|---|---|---|---|---|
| Kogashiwa et al, 2011 | 55 | M | Lower limb weakness[a]<br>ALS[b]<br>Lower limb weakness aggravated at the time of cancer manifestation with dysphagia and lymph node infiltration. | | | Hypopharyngeal squamous cell carcinoma, T3N2bM0 (+3 years) | | Response to tumor treatment with chemotherapy and radiotherapy (13-month follow-up). |
| Flanagan et al, 2012 | 31 | F | Left upper/lower limb weakness[a]<br>Lower MND[b]<br>*Rapidly progressive*<br>Spine MRI: signal alterations in cervical spinal cord ventral horns, enlargement and robust contrast enhancement of ventral roots in lumbar spine (sparing of dorsal roots). | | ↑ Protein | Hodgkin lymphoma (concurrently) | Not detected (serum and CSF) | Strong and persistent response to lymphoma treatment and IVIG |
| Psychogios et al, 2012 | 63 | M | Lower MND[a]<br>Lower MND (spinal)[b] | Behaviour/ personality disorder, dysautonomia, sensory axonal polyneuropathy | ↑ Cell count<br>↑ Protein<br>OCB(+) | SCLC<br>(PET-CT, biopsy) | Anti-Hu | Stabilization following antineoplastic and immune treatment (steroids, IVIG) |
| Lee et al, 2013 | 32 | F | Brachial amyotrophic diplegia[a]<br>Lower MND[b] | Dysautonomia | OCB(+) | Not detected (including with PET-CT) | Anti-Hu (serum and CSF) | No response to immune treatment (i.v. steroids, IVIG, PLEX, cyclophosphamide). Respiratory failure, died 11 months after symptom onset. |
| Mehrpour et al, 2013 | 79 | F | Pseudobulbar/bulbar[a]<br>ALS[b] | | | Gastric neuroendocrine tumor (+2 months) | Not detected (negative anti-Hu, -Ri,<br>-Yo) | Not reported |
| Younger et al, 2013 | 49 | F | Left lower limb[a]<br>ALS[b] | Cerebellar syndrome, startle myoclonus, cataplexy | Normal | Breast ductal carcinoma<br>(+3 months) | Anti-Ri | No response to IVIG or tumor therapy. Response to PLEX, significant decrease in anti-Ri ab titer. |
| Martinez et al, 2013 | 48 | F | Lower limbs, dysarthria[a]<br>PLS[b] | | Normal | Lung adenocarcinoma metastatic (-9 months) | Not detected | Parallel progression of cancer and MND. Died six months after MND onset. |
| Kacem et al, 2013 | 53 mean, 6 pts | | ALS[b] | | | Breast cancer (1)<br>Lung cancer (1) | Onconeural abs detected in all patients (6/6) | |
| Geevasinga | 66 | M | Bulbar, cognition (memory | | Normal | Lung adenocarcinoma | Anti- | No response to |

| | | | | | | | | |
|---|---|---|---|---|---|---|---|---|
| et al, 2014 | | | deficit), behavior disorder[a]<br>*C9ORF72*-positive FTD-ALS familial (bulbar, upper limbs)[b] | | | (+2 years, at MND progression) | Ma2/Ta<br>Anti-AChR | PLEX, progressive course |
| Spataro and La Bella, 2014 | 60 | F | Lower limbs, dysarthria[a]<br>Predominant upper MND[b]<br>*Rapidly progressive* | | Normal | Breast ductal cancer, sentinel axillary lymph nodes<br>(+ 10 months) | Not detected (Anti-Hu, -Ri,<br>-Yo: negative) | Strong and persistent response to cancer treatment |
| Diard-Detoeuf et al, 2014 | 80 | F | Upper limbs (bilaterally)[a]<br>Lower MND[b]<br>*Rapidly progressive*<br>Familial MND<br>*SOD1 118del* (heterozygous) | | ↑ Protein | Breast adenocarcinoma<br>(- 4 years) | Anti-Ri (serum) | Response to immune treatment (IVIG, oral prednisone) and cancer treatment |
| Struck et al, 2014 | 70 | M | Abdominal and limb fasciculations[a]<br>Lower MND[b]<br>**Autopsy-proven:**<br>MN loss and gliosis in ventral horns, mild MN loss in brainstem nuclei V and XII, Bunina bodies and ubiquitin-positive inclusions in surviving ventral horn MNs, atrophic ventral roots, normal-appearing CST, spared dorsal roots, no neuronal loss in frontal cortex, diaphragm atrophy. | Axonal sensory polyneuropathy | Normal | Non-Hodgkin lymphoma (follicular)<br>(-4 months) | Anti-β-tubulin IgM<br>Anti-GM1 IgM | No persistent response to lymphoma or immune treatment (IVIG, PLEX). Progressive course, died of aspiration pneumonia, respiratory failure. |
| Pillainayagam et al, 2015 | 59 | F | ALS[a]<br>ALS[b] (UMN and LMN signs)<br>*Rapidly progressive*<br>**Familial MND**<br>*History of poliomyelitis* | | Normal | Breast cancer (recurrence, (concurrently) | Anti-AChR | Partial response to IVIG |
| Verschueren et al., 2015 | 81 | F | Upper limbs>>lower limbs[a]<br>Lower MND (spinal)[b]<br>*Rapidly progressive* | | Normal | Malignant thymoma (B2) (+ months) | Anti-CV2/ CRMP5 | Improvement after tumor excision and immune treatment (3 IVIG courses, oral prednisone, azathioprine). At 2-year follow-up, persistent changes in distal upper limbs only. |
| | 48 | F | Lower limbs[a]<br>Lower MND (spinal)[b]<br>*Rapidly progressive* | | OCB(+) | Breast ductal adenocarcinoma<br>(+ 2 months) | Not detected | Stabilization after cancer and immune treatment (IVIG, oral prednisone, azathioprine, Rituximab). Restricted to lower limbs at four-year follow-up. |
| | 66 | F | Upper limbs[a] | | OCB(+) | Not detected | Anti-Hu | No response to |

| | | | | | | | | |
|---|---|---|---|---|---|---|---|---|
| | | | Lower MND[b]<br>*Rapidly progressive* | | | (including with PET-CT) | (serum) | immune treatment (oral steroids, cyclophosphamide). Progressive course, died of respiratory failure five months after MND onset. |
| Erdener et al, 2016 | 56 | M | Upper limbs (brachial diparesis)[a]<br>Lower MND (C5-C8, T1)[b] | Sensory neuronopathy | OCB(+) | SCLC (concurrently) | Anti-Hu | Stabilization with antineoplastic and immune treatment (steroids i.v.). Died 18 months after diagnosis. |
| Rosine et al, 2017 | 75 | M | ALS[a]<br>ALS[b] | Cerebellar syndrome | Normal | Prostatic adenocarcinoma (concurrently) | Anti-Yo (serum) | Partial response to cancer treatment and immune therapy (IVIG) |
| Riahi et al, 2017 | 5 pts 54.6 (mean) | | ALS[a]<br>ALS[b] | | Normal (5/5) | Colon adeno-carcinoma (2)<br>Lung cancer (1)<br>Gastric cancer (1)<br>Not detected (1, anti-Ri+) | Positive in 3/5:<br>Anti-Yo (2)<br>Anti-Ri (1) | No response to cancer excision (5/5) |
| Suzuki et al, 2018 | 75 | M | Lower limbs (R>L)[a]<br>Lower MND (spinal, bulbar)[b]<br>**Autopsy-proven:**<br>Severe MN loss and mild astrocytosis in ventral horns, hypoglossal nuclei degeneration,TDP-43 cytoplasmic inclusions in MN and astrocytes, perivascular B lymphocytes in spinal cord and surrounding anterior and posterior roots, inflammation and group atrophy in iliopsoas, unilateral gracile fasciculus degeneration, frontal lobe atrophy. | Sensorimotor polyneuropathy | ↑ Protein | Waldenström's macroglobulinemia | Monoclonal IgM $\kappa$ | Progressive course.<br>Died of respiratory failure, aspiration pneumonia. |
| Diamanti et al, 2018 | 44 | F | Upper and lower limbs (distal-to-proximal)[a]<br>Lower MND[b] | | OCB(+) | HER2+ breast cancer (concurrently).<br>Two small brain metastatic lesions (cerebellum, frontal). | Not detected (serum and CSF) | No response to cancer treatment or to steroids. Died of respiratory failure. |
| Mélé et al, 2018 | 64 | F | Upper limbs[a]<br>Lower MND[b] | Sensory neuronopathy | ↑ Protein<br>OCB(+) | Metastatic breast cancer<br>(-12 months) | Anti-Hu | No response to IVIG, cyclophosphamide. Progressive course. |
| | 64 | M | Upper limbs, tetrapyramidal, diaphragmatic paralysis[a]<br>ALS[b] | | ↑ Protein<br>OCB(-) | Squamous lung cancer (at relapse) | Anti-Hu | No response to IVIG. Cancer progression, death. |
| | 60 | M | Lower limbs[a]<br>Lower MND[b] | | ↑ Cell count | SCLC<br>(+3 months) | Anti-Hu | Stabilization with cancer and |

| | | | | | | | | |
|---|---|---|---|---|---|---|---|---|
| | | | | | ↑ Protein<br>OCB(-) | | | immune treatment (IVIG) |
| | 59 | M | Upper limbs[a]<br>Lower MND[b] | | ↑ Protein<br>OCB(-) | SCLC<br>(+2 months) | Anti-Hu | Improvement with cancer and immune treatment (IVIG, corticosteroids) |
| | 59 | M | Upper limbs[a]<br>Lower MND[b] | Sensory neuronopathy | Normal | None | Anti-Hu | Stabilization with immune treatment (IVIG) |
| | 59 | M | Upper limbs, tetrapyramidal[a]<br>ALS[b] | Limbic encephalitis | ↑ Protein<br>OCB(+) | SCLC (+6 months) | Anti-Hu | Stabilization with cancer and immune treatment (IVIG, cyclophosphamide). |
| | 61 | F | Upper limbs, diaphragmatic paralysis, bulbar[a]<br>Lower MND[b] | | ↑ Cell count<br>↑ Protein<br>OCB(+) | Squamous lung cancer (-4 months) | Anti-Hu | Stabilization with cancer and immune treatment (IVIG, corticosteroids) |
| Liu et al, 2019 | 59 | F | ALS[a]<br>ALS[b] | | ↑ Protein<br>OCB(+) | None | Anti-Yo | Patient denied treatment, progressive course, died of respiratory failure |
| | 55 | M | ALS[a]<br>ALS[b] | | Normal | None | Anti-Yo | Patient denied treatment, progressive course, died of aspiration |
| | 70 | M | ALS/PMA[a]<br>ALS/PMA[b] | | OCB(+) | None | Anti-Yo | Patient denied treatment, slowly progressive |
| | 53 | F | ALS[a]<br>ALS[b] | | Normal | None | Anti-Hu | Patient denied treatment, lost to follow-up |
| | 64 | M | ALS[a]<br>ALS[b] | Ataxia | Normal | None | Anti-Hu | Patient denied treatment, progressive course, died of pulmonary infection |
| | 59 | F | ALS[a]<br>ALS[b] | | | None | Anti-Ri | Patient denied treatment, lost to follow-up |
| | 56 | F | Upper limb weakness[a]<br>ALS/PMA[b] | | Normal | None | Anti-Ma2 | Patient denied treatment, Improvement |
| | 60 | M | Lower limb weakness[a]<br>ALS[b] | | Normal | None | Anti-Yo | Patient denied treatment, progressive course |
| | 32 | M | PMA[a]<br>PMA[b] | | ↑ Protein<br>OCB(+) | Papillary thyroid carcinoma | Not detected | Response to tumor treatment (surgery and $I^{131}$) |
| Goodfellow et al, 2019 | 61 | F | Lower limbs[a]<br>ALS[b] | | | Invasive ductal breast cancer | Not detected | No response to tumor |

| | | | | | | | | |
|---|---|---|---|---|---|---|---|---|
| | | | | | | | | treatment. Died 26 months after MND onset |
| | 57 | F | Right upper limb[a]<br>ALS[b]<br>*Rapidly progressive* | | | SCLC | Anti-Hu<br>Anti-CV2 | Response to tumor treatment (24-month follow-up) |
| Cheli et al, 2019 | 63 | M | ALS (upper/lower limbs)[a]<br>ALS (spinal)[b] | Limbic encephalitis (concurrently with SCLC) | ↑ Protein<br>OCB(+)<br>(type 3) | SCLC<br>(diagnosis -9 months, relapse 0 months) | Anti-Hu (serum and CSF) | Response to IVIG and tumor treatment |
| La Bella et al, 2019 | 79 | M | Right upper limb (proximally)[a]<br>Lower MND (spinal)[b]<br>*Rapidly progressive* | | 1st: Normal<br>2nd: ↑ Cell count (70% NK)<br>↑ Protein | Natural killer cells leukemia<br>(+4 months) | Not detected (Hu, Yo, Ri, gangliosides) | Transient response to antineoplastic treatment, died five months after MND onset |
| Pinto et al, 2019 | 40 | F | Quadriparesis, weight loss[a]<br>Lower MND (spinal)[b]<br>*Rapidly progressive* | Acanthosis nigricans | | Papillary urothelial bladder carcinoma | Anti-Hu (serum) | Strong response to cancer excision and immune therapy (IVIG, methylprednisolone) and amelioration of acanthosis nigricans. |
| Vogrig et al, 2019 | 59 | M | Lower limbs, bulbar[a]<br>ALS[b] | Encephalitis | OCB(+) | Not detected | Anti-Ma2 (serum and CSF) | Partial response to cyclophosphamide |
| | 56 | M | Lower limbs, bulbar[a]<br>Lower MND[b] | Encephalitis | ↑ Protein<br>OCB(+) | Germ cell tumor | Anti-Ma2 (serum and CSF) | Partial response to cyclophosphamide |
| | 47 | M | ALS[a]<br>ALS[b] | Encephalitis | OCB(+) | Testicular germ cell tumor (-1 year) | Anti-Ma2 (serum) | Response to cyclophosphamide and Rituximab. |
| Jaffer et al, 2020 | 63 | M | ALS[a]<br>ALS[b]<br>Immune checkpoint inhibitor-induced rapid worsening (Ipilimumab/ Nivolumab) | Myasthenia | Normal | Melanoma (metastatic) | Not detected | Progressive course.<br>No marked response to IVIG, PLEX.<br>Died of hypoxic respiratory failure, septic shock. |
| Tolkovsky et al, 2021 | 72 | M | Limb weakness,dysphagia, bilateral vocal cord paralysis[a]<br>ALS[b] | Dysautonomia, cerebellar ataxia, distal axonal sensorimotor polyneuropathy | ↑ Cell count<br>↑ Protein<br>OCB(+) | Not detected (including with PET-CT) | Anti-Hu<br>Anti-CV2/ CRMP5 | Stabilization after i.v. methylprednisolone and cyclophosphamide (15-month follow-up) |
| Riku et al, 2021 | 68 | M | Limb weakness[a]<br>ALS[b]<br>**Autopsy-proven:**<br>Betz cell loss and astrogliosis in motor cortex, CST | Gaze palsy, opsoclonus | ↑ Protein | Prostatic small cell carcinoma (concurrently) | Anti-Yo | Partial improvement following IVIG. Died of respiratory |

| | | | | | | | |
|---|---|---|---|---|---|---|---|
| | | | degeneration, abundant CD68+ foamy macrophages in CST, MN loss in ventral horns, ventral root atrophy, grouped muscle atrophy. | | | | failure and sepsis 36 months after neurological symptom onset. |
| Akan and Baysal-Kirac, 2021 | 78 | M | Lower limbs[a]<br>Lower MND[b] | | Prostate adenocarcinoma (concurrently) | Not tested | No response to tumor resection. Died nine months after MND diagnosis. |
| | 84 | F | Bulbar, Right upper limb[a]<br>ALS[b] | | Endometrial serous adenocarcinoma (-20 months) | Not tested | RT, complete cancer remission. Died nine months after MND diagnosis. |
| | 61 | M | Left upper limb[a]<br>ALS[b] | | NSCLC (adenocarcinoma) with lymph node infiltration (+12 months)<br>Colorectal adenoma (years ago) | Not tested | No response to RT, CT.<br>Died eight months after MND diagnosis |
| | 66 | M | Right lower limb[a]<br>ALS[b] | | Laryngeal squamous cell carcinoma (-24 months) | Not tested | Surgery/RT, complete cancer remission. Died two years after MND diagnosis. |
| | 45 | M | Right lower limb[a]<br>Lower MND (spinal)[b] | Normal | NSCLC with lymph node infiltration (+3 years) | Not tested | Stabilization for one year after tumor resection, RT, CT, IVIG |
| Shiba et al, 2021 | 69 | F | Lower limbs[a]<br>ALS[b] | | Mesenteric lymphoma (-2 months) | | Died 9 months after ALS onset |
| Kleinschmidt-DeMasters et al, 2021 | 77 | F | Left upper and lower limbs[a]<br>Lower MND (spinal)[b]<br>**Autopsy-proven:**<br>Ventral horn MN loss (mostly lower thoracic and lumbar), lymphocytic infiltrates (perivascular, parenchymal), CD3+ T lymphocytes, diffuse microgliosis (CD68), moderate astrocytosis, mostly in ventral horns and slightly in spinal cord white matter. | ↑ Cell count | Hodgkin lymphoma (-2 months) | | No response to steroids, IVIG, PLEX. Died five months after MND onset (respiratory failure). |
| Rocha et al, 2021 | 55 | F | ALS[a]<br>ALS[b] | | Invasive ductal breast cancer, BRCA2+ (relapse, -1 month) | Anti-Ma2 | No neurologic response to mastectomy and HT. Patient denied CT. She died one year later. |
| | 48 | M | Lower MND[a]<br>Lower MND[b]<br>*Rapidly progressive* | | Thyroid Hurthle cancer (+13 months) | Anti-Yo | Initial stabilization for eight months with IVIG and hemithyroide-ctomy. Secondary MND progression. |

| | | | | | | | | |
|---|---|---|---|---|---|---|---|---|
| | 74 | F | ALS[a]<br>ALS[b]<br>*Rapidly progressive* | | | Invasive ductal breast cancer<br>(cancer, 2014; MND, 01/2016) | Not tested | Died 13 months after MND onset. Cancer treated with surgery, RT, HT. |
| | 77 | M | ALS[a]<br>ALS[b] | | | Lung adenocarcinoma<br>(+1 year) | Not tested | Died six years after MND onset |
| Yang et al, 2022 | 49 | M | Right upper limb[a]<br>ALS[b] | | Not reported | No tumor detected (including with PET-CT) | SOX1, GAD65 | No response to IVIG. Died of respiratory failure one year later. |
| | 46 | M | Left lower limb[a]<br>ALS[b] | | ↑ Protein | Renal clear cell carcinoma<br>(+2 months) | Not detected (serum, CSF) | Partial response to IVIG, prednisolone, tumor resection. Stable at one-year follow-up. |
| | 48 | M | Lower limbs[a]<br>ALS[b] | | ↑ Cell count<br>↑ Protein | Chronic myelogenous leukemia<br>(+6 months) | Not detected (serum, CSF) | Partial response to methylprednisolone. Stable at one-year follow-up. *Repeat CSF analysis revealed significant decrease in cell count and protein levels.* |
| Echefu et al, 2023 | 60 | M | Lower>>upper limbs[a]<br>ALS[b] | | | Pheochromocytoma<br>(+2 months) | Negative GAD-65 and amphiphysin, patient refused further testing | Stabilization after tumor resection (18-month follow-up) |
| Vadell and Miralles, 2024 | 63 | F | Upper limbs[a]<br>Lower MND (spinal)[b] | Dysautonomia | Normal | Lung cancer<br>(+7 months) | Anti-Hu<br>ANA | No response to IVIG, progressive course. Died 19 months after symptom onset. |
| This study | 51 | F | Right upper limb[a]<br>ALS[b] | | Normal | Invasive ductal breast cancer<br>(+1 year) | Not detected | Partial response to immune therapy (i.v. steroids, IVIG). Died of pulmonary embolism 31 months after MND symptom onset. |
| | 71 | M | Left lower limb[a]<br>ALS[b] | Cerebellar syndrome | ↑ Protein<br>↑ Albumin index | Squamous lung carcinoma<br>(+3 years) | SOX1 | Progressive course. Died four years after MND symptom onset. |
| | 63 | M | Lower limbs[a]<br>ALS[b] | | Normal | Urinary bladder cancer (-12 months) | SOX1 | No response to cancer or immune |

| | | | | | | | |
|---|---|---|---|---|---|---|---|
| | | | | | | | treatment (IVIG, i.v. steroids, cyclophosphamide). |
| 71 | F | Dysarthria (bulbar)[a] ALS[b] | Cerebellar, dysautonomia | Normal | Neuroendocrine SCLC (+22 months) | Not detected | Partial response to IVIG with amelioration of dysphagia, dysarthria and cerebellar dysfunction. Partial response following tumor's surgical excision. |

**Footnotes:** Before MND onset, - x months or years; After MND onset, + x months or years; Age, Age at MND onset; Sex, biological sex assigned at birth.
**Abbreviations:** pts, patients; MND, motor neuron disease; ALS, amyotrophic lateral sclerosis; PMA, progressive muscular atrophy; MN, motor neurons; CSF, cerebrospinal fluid; OCB, oligoclonal bands; SCLC, small cell lung cancer. NSCLC, non-small cell lung cancer; MGUS, monoclonal gammopathy of unknown significance; SOX-1, sry-like high mobility group box 1; GAD65, glutamic acid decarboxylase 65; ANA, Anti-nuclear antibodies; IVIG, intravenous immunoglobulin; PLEX, plasma exchange; CT, chemotherapy; HT, hormone therapy; RT, radiotherapy.

**Supplementary Table 1. Clinical and laboratory data of paraneoplastic motor neuron disease case studies.**

Published cases of MND associated with neoplastic disease are presented in chronological order from the past to present along with deep clinical and laboratory data, as these are reported in published articles. The data variables (or items) collected for each case include patient's sex (biological), age at MND onset, site of MND onset, MND phenotype, concurrent neurologic paraneoplastic syndromes or other neurologic non-paraneoplastic syndromes or non-neurologic paraneoplastic syndromes, cerebrospinal fluid analysis, the associated neoplastic disease and its temporal association with MND symptom onset, onconeural or other antibodies expressed, the response to antineoplastic or immune treatment and the outcome. A subset of cases also includes autopsy and histologic examination of the nervous system, which is reported concisely, and some also include genetic testing for MND-causative genetic deficits. The temporal association of neoplasm diagnosis with MND symptom onset is referred to as – x months or years when the neoplasm is diagnosed before MND onset, or as + x months or years when the neoplasm is diagnosed after MND symptom onset. The normal CSF refers to CSF with cell count and protein in the reference range and absence of oligoclonal bands. Note that certain data variables are not known for a subset of cases. Footnotes and abbreviations are stated at the bottom of the table.

**Supplementary Table 2. PNS-Care-based diagnostic evaluation of paraneoplastic motor neuron disease cases.**

| Case study | Subject | Clinical level | Laboratory level | Neoplasm | Total score | Diagnostic level | Response |
|---|---|---|---|---|---|---|---|
| Henson et al, 1954 | 1 | 3 | 0 | 4 | 7 | Probable | |
| | 2 | 2 | 0 | 4 | 6 | Probable | |
| Rowland and Schneck, 1963 | 1 | 2 | 0 | 4 | 6 | Probable | |
| | 2 | 2 | 0 | 4 | 6 | Probable | |
| Brain et al, 1965 | 1 | 2 | 0 | 4 | 6 | Probable | |
| | 2 | 3 | 0 | 4 | 7 | Probable | Response |
| Walton et al, 1968 | | 2 | 0 | 4 | 6 | Probable | |
| Adams et al, 1970 | | 2 | 0 | 4 | 6 | Probable | |
| Buchanan and Malamud, 1973 | | 2 | 0 | 4 | 6 | Probable | Response |
| Smith et al, 1975 | | 2 | 0 | 4 | 6 | Probable | |
| Bauer et al, 1977 | | 2 | 0 | 4 | 6 | Probable | |
| Peacock et al, 1979 | | 2 | 0 | 4 | 6 | Probable | Response |
| Mitchell and Olczak, 1979 | | 2 | 0 | 4 | 6 | Probable | Response |
| Gritzman et al, 1983 | 1 | 2 | 0 | 4 | 6 | Probable | |
| | 2 | 2 | 0 | 4 | 6 | Probable | |
| Thomas et al, 1984 | | 2 | 0 | 4 | 6 | Probable | |
| Recine et al, 1984 | | 2 | 0 | 4 | 6 | Probable | |
| Stoll et al, 1984 | | 2 | 0 | 4 | 6 | Probable | |
| Evans et al, 1990 | | 2 | 0 | 4 | 6 | Probable | Response |
| Hays et al, 1990 | | 2 | 0 | 4 | 6 | Probable | |
| Younger et al, 1991 | 1 | 2 | 0 | 4 | 6 | Probable | |
| | 2 | 2 | 0 | 4 | 6 | Probable | |
| | 3 | 2 | 0 | 4 | 6 | Probable | |
| Dalmau et al, 1992 | 1 | 2 | 3 | 4 | 9 | Definite | |
| | 2 | 3 | 3 | 4 | 10 | Definite | |
| | 3 | 3 | 3 | 4 | 10 | Definite | |
| Rowland et al, | | 2 | 0 | 4 | 6 | Probable | |

| | | | | | | | |
|---|---|---|---|---|---|---|---|
| 1995 | | | | | | | |
| Verma et al, 1996 | | 2 | 3 | 4 | 9 | Definite | |
| Forsyth et al, 1997 | 1 | 2 | 0 | 4 | 6 | Probable | |
| | 2 | 2 | 0 | 4 | 6 | Probable | |
| | 3 | 2 | 0 | 4 | 6 | Probable | |
| | 4 | 2 | 0 | 4 | 6 | Probable | |
| | 5 | 2 | 0 | 4 | 6 | Probable | |
| | 6 | 2 | 0 | 4 | 6 | Probable | |
| | 7 | 2 | 0 | 4 | 6 | Probable | |
| Herrero et al, 1998 | | 2 | 0 | 4 | 6 | Probable | |
| Khwaja et al, 1998 | | 2 | 3 | 4 | 9 | Definite | |
| Correa et al, 1999 | | 2 | 0 | 4 | 6 | Probable | |
| Forman et al, 1999 | | 2 | 0 | 4 | 6 | Probable | Response |
| Ferracci et al, 1999 | | 2 | 0 | 4 | 6 | Probable | Partial response |
| Rijnders and Decramer, 2000 | | 2 | 0 | 4 | 6 | Probable | Partial response |
| Vigliani et al, 2000 | 14 pts | 2 | 0 | 4 | 6 | Probable (14 pts) | |
| Bir et al, 2000 | | 2 | 0 | 4 | 6 | Probable | Response |
| Viera-Alemán et al, 2002 | | 2 | 0 | 4 | 6 | Probable | |
| Khealani et al, 2004 | | 3 | 0 | 4 | 7 | Probable | |
| Ogawa et al, 2004 | | 3 | 3 | 4 | 10 | Definite | |
| Jeon et al, 2004 | | 2 | 0 | 4 | 6 | Probable | |
| Chang et al, 2004 | | 2 | 3 | 4 | 9 | Definite | Response |
| Kijima et al, 2005 | | 2 | 0 | 4 | 6 | Probable | Response |
| Gazic et al, 2005 | | 3 | 3 | 4 | 10 | Definite | |
| Hays et al, 2006 | | 2 | 0 | 4 | 6 | Probable | |
| Waragai et al, 2006 | | 3 | 3 | 4 | 10 | Definite | Response |
| Kararizou et al, 2007 | | 2 | 0 | 4 | 6 | Probable | Response |
| Sadot et al, 2007 | 1 | 2 | 0 | 4 | 6 | Probable | |
| | 2 | 2 | 0 | 4 | 6 | Probable | |

| | | | | | | | |
|---|---|---|---|---|---|---|---|
| Turgut et al, 2007 | | 2 | 0 | 4 | 6 | Probable | |
| Sato et al, 2007 | | 2 | 0 | 4 | 6 | Probable | |
| Cánovas et al, 2007 | | 2 | 0 | 4 | 6 | Probable | Response |
| Martín et al, 2007 | | 2 | 3 | 0 | 5 | Possible | |
| Hoffmann et al, 2008 | | 2 | 3 | 0 | 5 | Possible | |
| Tofaris and Farmer, 2008 | | 2 | 3 | 4 | 9 | Definite | |
| Koc and Yerdelen, 2008 | | 2 | 0 | 4 | 6 | Probable | |
| Turk et al, 2009 | | 2 | 0 | 4 | 6 | Probable | Response |
| Distad and Weiss, 2010 | | 3 | 3 | 4 | 10 | Definite | |
| Ducray et al, 2010 | 1 | 2 | 3 | 4 | 9 | Definite | Response |
| | 2 | 3 | 3 | 4 | 10 | Definite | |
| | 3 | 2 | 3 | 4 | 9 | Definite | Response |
| Briani et al, 2011 | 4 pts | 2 | 0 | 4 | 6 | Probable (4 pts) | |
| Jurici et al., 2011 | | 2 | 0 | 4 | 6 | Probable | |
| Piccolo et al., 2011 | | 2 | 3 | 0 | 5 | Possible | |
| Kogashiwa et al, 2011 | | 2 | 0 | 4 | 6 | Probable | Response |
| Flanagan et al, 2012 | | 2 | 0 | 4 | 6 | Probable | Response |
| Psychogios et al, 2012 | | 2 | 3 | 4 | 9 | Definite | Response |
| Lee et al, 2013 | | 2 | 3 | 1 | 6 | Probable | |
| Mehrpour et al, 2013 | | 2 | 0 | 4 | 6 | Probable | |
| Younger et al, 2013 | | 3 | 3 | 4 | 10 | Definite | Response |
| Martinez et al, 2013 | | 2 | 0 | 4 | 6 | Probable | |
| Kacem et al, 2013 | 2 pts | 2 | 3 | 4 | 9 | Definite (2 pts) | |
| | 4 pts | 2 | 3 | 1 | 6 | Probable (4 pts) | |
| Geevasinga et al, 2014 | | 2 | 3 | 4 | 9 | Definite | |
| Spataro and La Bella, 2014 | | 2 | 0 | 4 | 6 | Probable | Response |

| | | | | | | | |
|---|---|---|---|---|---|---|---|
| Diard-Detoeuf et al, 2014 | | 2 | 3 | 4 | 9 | Definite | Response |
| Struck et al, 2014 | | 2 | 0 | 4 | 6 | Probable | |
| Pillainayagam et al, 2015 | | 2 | 0 | 4 | 6 | Probable | Partial response |
| Verschueren et al., 2015 | 1 | 2 | 3 | 4 | 9 | Definite | Response |
| | 2 | 2 | 0 | 4 | 6 | Probable | Response |
| | 3 | 2 | 3 | 1 | 6 | Probable | |
| Erdener et al, 2016 | | 3 | 3 | 4 | 10 | Definite | Response |
| Rosine et al, 2017 | | 3 | 3 | 4 | 10 | Definite | Response |
| Riahi et al, 2017 | 2 pts | 2 | 3 | 4 | 9 | Definite (2 pts) | |
| | 2 pts | 2 | 0 | 4 | 6 | Probable (2 pts) | |
| | 1 pt | 2 | 3 | 1 | 6 | Probable (1 pt) | |
| Suzuki et al, 2018 | | 2 | 0 | 4 | 6 | Probable | |
| Diamanti et al, 2018 | | 2 | 0 | 4 | 6 | Probable | |
| Mélé et al, 2018 | 1 | 3 | 3 | 4 | 10 | Definite | |
| | 2 | 2 | 3 | 4 | 9 | Definite | |
| | 3 | 2 | 3 | 4 | 9 | Definite | Response |
| | 4 | 2 | 3 | 4 | 9 | Definite | Response |
| | 5 | 3 | 3 | 1 | 7 | Probable | Response |
| | 6 | 3 | 3 | 4 | 10 | Definite | Response |
| | 7 | 2 | 3 | 4 | 9 | Definite | Response |
| Liu et al, 2019 | 1 | 2 | 3 | 1 | 6 | Probable | |
| | 2 | 2 | 3 | 1 | 6 | Probable | |
| | 3 | 2 | 3 | 1 | 6 | Probable | |
| | 4 | 2 | 3 | 1 | 6 | Probable | |
| | 5 | 2 | 3 | 1 | 6 | Probable | |
| | 6 | 2 | 3 | 1 | 6 | Probable | |
| | 7 | 2 | 3 | 1 | 6 | Probable | |
| | 8 | 2 | 3 | 1 | 6 | Probable | |
| | 9 | 2 | 0 | 4 | 6 | Probable | Response |
| Goodfellow et al, 2019 | 1 | 2 | 0 | 4 | 6 | Probable | |
| | 2 | 2 | 3 | 4 | 9 | Definite | Response |
| Cheli et al, 2019 | | 3 | 3 | 4 | 10 | Definite | Response |
| La Bella et al, 2019 | | 2 | 0 | 4 | 6 | Probable | |
| Pinto et al, 2019 | | 2 | 3 | 4 | 9 | Definite | Response |
| Vogrig et al, 2019 | 1 | 2 | 3 | 1 | 6 | Probable | Partial response |
| | 2 | 2 | 3 | 4 | 9 | Definite | Partial response |
| | 3 | 2 | 3 | 4 | 9 | Definite | Response |

| | | | | | | | |
|---|---|---|---|---|---|---|---|
| Jaffer et al, 2020 | | 2 | 0 | 4 | 6 | Probable | |
| Tolkovsky et al, 2021 | | 3 | 3 | 1 | 7 | Probable | Response |
| Riku et al, 2021 | | 3 | 3 | 4 | 10 | Definite | Response |
| Akan and Baysal-Kirac, 2021 | 1 | 2 | 0 | 4 | 6 | Probable | |
| | 2 | 2 | 0 | 4 | 6 | Probable | |
| | 3 | 2 | 0 | 4 | 6 | Probable | |
| | 4 | 2 | 0 | 4 | 6 | Probable | |
| | 5 | 2 | 0 | 4 | 6 | Probable | Response |
| Shiba et al, 2021 | | 2 | 0 | 4 | 6 | Probable | |
| Kleinschmidt-DeMasters et al, 2021 | | 2 | 0 | 4 | 6 | Probable | |
| Rocha et al, 2021 | 1 | 2 | 3 | 4 | 9 | Definite | |
| | 2 | 2 | 3 | 4 | 9 | Definite | |
| | 3 | 2 | 0 | 4 | 6 | Probable | |
| | 4 | 2 | 0 | 4 | 6 | Probable | |
| Yang et al, 2022 | 1 | 2 | 3 | 1 | 6 | Probable | |
| | 2 | 2 | 0 | 4 | 6 | Probable | Partial response |
| | 3 | 2 | 0 | 4 | 6 | Probable | Partial response |
| Echefu et al, 2023 | | 2 | 0 | 4 | 6 | Probable | Response |
| Vadell and Miralles, 2024 | | 2 | 3 | 4 | 9 | Definite | |
| This study | 1 | 2 | 0 | 4 | 6 | Probable | Response |
| | 2 | 3 | 3 | 4 | 10 | Definite | |
| | 3 | 2 | 3 | 4 | 9 | Definite | |
| | 4 | 3 | 0 | 4 | 7 | Probable | Response |

**Supplementary Table 2. PNS-Care-based diagnostic evaluation of paraneoplastic motor neuron disease cases.**

All cases of paraneoplastic MND reported in Supplementary Table 1 are evaluated for the level of diagnostic certainty for paraneoplastic MND according to the PNS-Care diagnostic criteria for paraneoplastic neurologic syndromes (Graus et al, 2021). Of 163 cases, 42 cases (26%) represent definite paraneoplastic MND, 118 cases (72%) represent probable paraneoplastic MND, and 3 cases (2%) represent possible paraneoplastic MND. Neurologic response to cancer and/or immune treatments is reported as a separate feature since it is not part of the PNS-Care score but it increases the diagnostic confidence especially in cases with a low PNS-Care score.

**Supplementary references**


1. Henson RA, Russell DS, Wilkinson M. Carcinomatous neuropathy and myopathy a clinical and pathological study. *Brain*. 1954;77(1):82-121. doi:10.1093/brain/77.1.82.
2. Rowland LP, Schneck SA. Neuromuscular disorders associated with malignant neoplastic disease. *J Chronic Dis*. 1963;16(7):777-795. doi:10.1016/0021-9681(63)90011-0.
3. Brain, Lord, Croft P, Wilkinson M. Motor neurone disease as a manifestation of neoplasm. *Brain*. 1965;88(3):479-500. doi:10.1093/brain/88.3.479.
4. Walton JN, Tomlinson BE, Pearce GW. Subacute "Poliomyelitis" and Hodgkin's Disease. *J Neurol Sci*. 1968;6:435-445.
5. Adams RD, Richardson EPJ, Castleman B. Case 42-1970-Progressive weakness and sensory loss affecting the neck and arms. *N Engl J Med*. 1970;283(15):806-814. doi:10.1056/NEJM197010082831512.
6. Buchanan DS, Malamud N. Motor neuron disease with renal cell carcinoma and postoperative neurologic remission: A clinicopathologic report. *Neurology*. 1973;23(8):891-894. doi:10.1212/wnl.23.8.891.
7. Smith TW, Tyler HR, Schoene WC. Atypical astrocytes and Rosenthal fibers in a case of amyotrophic lateral sclerosis associated with a cerebral glioblastoma multiforme. *Acta Neuropathol*. 1975;31(1):29-34. doi:10.1007/BF00696884.
8. Bauer M, Bergstrom R, Ritter B, Olsson Y. Macroglobulinemia Waldenstrom and motor neuron syndrome. *Acta Neurol Scand*. 1977;55:245-250.
9. Peacock A, Dawkins K, Rushworth G. Motor neurone disease associated with bronchial carcinoma? *Br Med J*. 1979:499-500.
10. Mitchell D, Olczak S. Remission of a syndrome indistinguishable from motor neurone disease after resection of bronchial carcinoma. *Br Med J*. 1979;2(6183):176-177. doi:10.1136/bmj.2.6183.176-a.
11. Gritzman MCD, Fritz VU, Perkins S, Kaplan CL. Motor neuron disease associated with carcinoma. A report of 2 cases. *South African Med J*. 1983;63(8):288-291.
12. Thomas NE, Passamonte PM, Sunderrajan E V., Andelin JB, Ansbacher LE. Bilateral diaphragmatic paralysis as a possible paraneoplastic syndrome from renal cell carcinoma. *Am Rev Respir Dis*. 1984;129(3):507-509.
13. Recine U, Longhi C, Pelosio A, Massini R. An unusually severe subacute motor neuronopathy in hodgkin's disease. *Acta Haematol*. 1984;71(2):135-138.

doi:10.1159/000206573.

14. Stoll DB, Lublin F, Brodovsky H, Frederick Laucius J, Patchefsky A, Cooper H. Association of subacute motor neuronopathy with thymoma. *Cancer*. 1984;54(4):770-772. doi:10.1002/1097-0142(1984)54:4<770::AID-CNCR2820540430>3.0.CO;2-E.
15. Evans B, Fagan C, Arnold T, Dropcho EJ, Oh S. Paraneoplastic motor neuron disease and renal cell carcinoma Improvement after nephrectomy. *Neurology*. 1990;40(6):99-104. doi:https://doi.org/10.1212/WNL.40.6.960.
16. Hays AP, Roxas A, Sadiq S, et al. A monoclonal IgA in a patient with amyotrophic lateral sclerosis reacts with neurofilaments and surface antigen on neuroblastoma cells. *J Neuropathol Exp Neurol*. 1990;49(4):383-398.
17. Younger DS, Rowland LP, Latov N, et al. Lymphoma, motor neuron diseases, lateral sclerosis. *Ann Neurol*. 1991;29:78-86.
18. Dalmau J, Graus F, Rosenblum MK, Posner JB. Anti-Hu-associated paraneoplastic encephalomyelitis/sensory neuronopathy. A clinical study of 71 patients. *Med*. 1992;71(2):59-72. doi:10.1097/00005792-199203000-00001.
19. Rowland LP, Sherman WL, Hays AP, Lange DJ, Latov N, Younger DS. Autopsy-proven amyotrophic lateral sclerosis, Waldenstrom's macroglobulinemia, and antibodies to sulfated glucuronic acid paragloboside. *Neurology*. 1995;45(April):827-829.
20. Verma A, Berger JR, Snodgrass S, Petito C. Motor neuron disease: A paraneoplastic process associated with anti-Hu antibody and small-cell lung carcinoma. *Ann Neurol*. 1996;40(1):112-116. doi:10.1002/ana.410400118.
21. Forsyth PA, Dalmau J, Graus F, Cwik V, Rosenblum MK, Posner JB. Motor neuron syndromes in cancer patients. *Ann Neurol*. 1997;41(6):722-730. doi:10.1002/ana.410410608.
22. Herrero S, Cantalapiedra A, Perez-Oteyza J, Bellas C, Gobernado J, Odriozola J. Mantle cell lymphoma with amyotrophic lateral sclerosis (motor neuron disease). *Haematologica*. 1998;83(4):382-383. http://www.ncbi.nlm.nih.gov/entrez/query.fcgi?cmd=Retrieve&db=PubMed&dopt=Citation&list_uids=16963398.
23. Khwaja S, Sripathi N, Ahmad BK, Lennon VA. Paraneoplastic motor neuron disease with type 1 Purkinje cell antibodies. *Muscle and Nerve*. 1998;21(7):943-945. doi:10.1002/(SICI)1097-4598(199807)21:7<943::AID-MUS14>3.0.CO;2-R.
24. Correa PS, Benito AY, Ortega SS, Esmenota MJ, Rivero P, Diaz C. [Sweet's syndrome and motor neuron disease associated with esophageal carcinoma]. *Case Reports An Med Interna*.

1999;16(8):423-426.

25. Forman D, Rae-Grant AD, Matchett SC, Cowen JS. A reversible cause of hypercapnic respiratory failure: Lower motor neuronopathy associated with renal cell carcinoma. *Chest*. 1999;115(3):899-901. doi:10.1378/chest.115.3.899.
26. Ferracci F, Fassetta G, Butler MH, Floyd S, Solimena M, De Camilli P. A novel antineuronal antibody in a motor neuron syndrome associated with breast cancer. *Neurology*. 1999;53(4):852-855. doi:10.1212/wnl.53.4.852.
27. Berghs S, Ferracci F, Maksimova E, et al. Autoimmunity to βIV spectrin in paraneoplastic lower motor neuron syndrome. *Proc Natl Acad Sci U S A*. 2001;98(12):6945-6950. doi:10.1073/pnas.121170798.
28. Rijnders B, Decramer M. Reversibility of paraneoplastic bilateral diaphragmatic paralysis after nephrectomy for renal cell carcinoma. *Ann Oncol*. 2000;11:221-225. https://doi.org/10.1023/A:1008382030802.
29. Vigliani MC, Polo P, Chio A, Giometto B, Mazzini L, Schiffer D. Patients with amyotrophic lateral sclerosis and cancer do not differ clinically from patients with sporadic amyotrophic lateral sclerosis. *J Neurol*. 2000;247:778-782.
30. Bir LS, Keskin A, Yaren A, Sermez Y, Oguzhanoglu A, Şahiner T. Lower motor neuron disease associated with myelofibrosis. *Clin Neurol Neurosurg*. 2000;102(2):109-112. doi:10.1016/S0303-8467(00)00072-X.
31. Viera-Alemán C, Neninger-Vinageras E, I Pedro-Silva, et al. Hypernephroma associated motor neuron disease. Presentation of a case. *Rev Neurol*. 2002;34(11):1044-1048.
32. Khealani BA, Qureshi R, Wasay M. Motor euronopathy associated with adenocarcinoma of esophagus. *J Pak Med Assoc*. 2004;54(3):165-166.
33. Ogawa M, Nishie M, Kurahashi K, Kaimori M, Wakabayashi K. Anti-Hu associated paraneoplastic sensory neuronopathy with upper motor neurone involvement. *J Neurol Neurosurg Psychiatry*. 2004;75(7):1051-1053. doi:10.1136/jnnp.2003.024265.
34. Jeon SB, Lim Y, Kim K, Ph D. A case of lower motor neuron syndrome which may be paraneoplastic. *J Korean Neurol Assoc*. 2004;22(3):265-268.
35. Chang CY, Martinu T, Witsell DL. Bilateral vocal cord paresis as a presenting sign of paraneoplastic syndrome: Case report. *Otolaryngol - Head Neck Surg*. 2004;130(6):788-790. doi:10.1016/S0194-5998(03)01319-6.
36. Kijima Y, Yoshinaka H, Higuchi I, Owaki T, Aikou T. A case of amyotrophic lateral sclerosis and breast cancer. *Breast Cancer*. 2005;12(1):57-59. doi:10.2325/jbcs.12.57

37. Gazic B, Pizem J, Dolenc-Groselj L, Svigelj V, Popovic M. Paraneoplastic encephalomyelitis/sensory motor peripheral neuropathy - An autopsy case study. *Folia Neuropathol*. 2005;43(2):113-117.
38. Hays AP, Naini A, He CZ, Mitsumoto H, Rowland LP. Sporadic amyotrophic lateral sclerosis and breast cancer: Hyaline conglomerate inclusions lead to identification of SOD1 mutation. *J Neurol Sci*. 2006;242(1-2 SPEC. ISS.):67-69. doi:10.1016/j.jns.2005.11.016.
39. Waragai M, Chiba A, Uchibori A, Fukushima T, Anno M, Tanaka K. Anti-Ma2 associated paraneoplastic neurological syndrome presenting as encephalitis and progressive muscular atrophy. *J Neurol Neurosurg Psychiatry*. 2006;77(1):111-113. doi:10.1136/jnnp.2005.068775.
40. Kararizou E, Stamboulis E, Markou I, Alevizaki M, Gkiatas K. Amyotrophic lateral sclerosis and prolactinoma. *Funct Neurol*. 2007;22(1):39-41.
41. Sadot E, Carluer L, Corcia P, Delozier Y, Levy C, Viader F. Breast cancer and motor neuron disease: Clinical study of seven cases. *Amyotroph Lateral Scler*. 2007;8(5):288-291. doi:10.1080/17482960701419505.
42. Turgut N, Karagöl H, Celik Y, Uygun K, Reyhani A. Subacute motor neuronopathy associated with hepatocellular carcinoma [2]. *J Neurooncol*. 2007;83(1):95-96. doi:10.1007/s11060-006-9296-9.
43. Sato Y, Takayama T, Nikaido T, et al. Report of an autopsy case of colon cancer with amyotrophic lateral sclerosis. *Japanese J Gastroenterol*. 2007;104(9):1365-1370.
44. Cánovas D, Martinez JM, Viguera M, Ribera G. [Association of renal carcinoma with neuromyotonia and involvement of inferior motor neuron]. *Neurologia*. 2007;22(6):399-400.
45. Martín GG, Errazquin FP, Munoz MC, Acebal MR. [Amyotrophic lateral sclerosis and anti-CV2 antibodies. Paraneoplastic association?]. *Neurologia*. 2007;22(6):406-409.
46. Hoffmann LA, Jarius S, Pellkofer HL, et al. Anti-Ma and anti-Ta associated paraneoplastic neurological syndromes: 22 Newly diagnosed patients and review of previous cases. *J Neurol Neurosurg Psychiatry*. 2008;79(7):767-773. doi:10.1136/jnnp.2007.118588.
47. Tofaris GK, Farmer SF. Focal Paraneoplastic Syndrome associated with small cell carcinoma of the lung [1]. *J Neurol*. 2008;255(1):123-124. doi:10.1007/s00415-008-0550-2
48. Koc F, Yerdelen D. Motor neuron disease and its association with non-Hodgkins lymphoma. *Neurosci*. 2008;13(4):458-459.
49. Turk HM, Ozet A, Kuzhan O, et al. Paraneoplastic motor neuron disease resembling amyotrophic lateral sclerosis in a patient with renal cell carcinoma. *Med Princ Pract*.

2009;18(1):73-75. doi:10.1159/000163053.

50. Distad BJ, Weiss MD. Paraneoplastic motor neuron disease associated with purkinje cell autoantibody type 1. *J Clin Neuromuscul Dis*. 2010;12(1):36-41. doi:10.1097/CND.0b013e3181c50528.
51. Ducray F, Graus F, Vigliani MC, et al. Delayed onset of a second paraneoplastic neurological syndrome in eight patients. *J Neurol Neurosurg Psychiatry*. 2010;81(8):937-939. doi:10.1136/jnnp.2009.190199.
52. Briani C, Vitaliani R, Grisold W, et al. Spectrum of paraneoplastic disease associated with lymphoma. *Neurology*. 2011;76(8):705-710. doi:10.1212/WNL.0b013e31820d62eb.
53. Jurici S, Laquerrière A, Bedat-Millet AL, et al. An autopsy case of amyotrophic lateral sclerosis with waldenström macroglobulinemia and anti-MAG gammopathy. *Case Rep Neurol*. 2011;3(3):294-300. doi:10.1159/000335004.
54. Piccolo G, Tavazzi E, Jarius S, et al. Anti-Ma2/Ta antibodies in a woman with primary lateral sclerosis-like phenotype and Sjögren syndrome. *Neurol Sci*. 2011;32(5):915-917. doi:10.1007/s10072-011-0510-y.
55. Kogashiwa Y, Oishi N, Yamauchi K, Kohno N. Advanced hypopharyngeal cancer with amyotrophic lateral sclerosis. *Auris Nasus Larynx*. 2011;38(6):750-752. doi:10.1016/j.anl.2011.01.005.
56. Flanagan EP, Sandroni P, Pittock SJ, Inwards DJ, Jones LK. Paraneoplastic lower motor neuronopathy associated with Hodgkin lymphoma. *Muscle and Nerve*. 2012;46(5):823-827. doi:10.1002/mus.23464.
57. Psychogios K, Markakis I, Athanasiadou K, Gekas G. Paraneoplastic neurological syndrome associated with occult small-cell lung carcinoma: tumor detection by FDG-PET. *Nevrologia* (*Archives Clin Neurol.*) 2012;21(4):29-38. https://www.jneurology.gr/ojs/index.php/aocn/article/view/24.
58. Lee JI, MacHt S, Albrecht P, Hartung HP, Goebels N. Brachial amyotrophic diparesis associated with anti-Hu positive anterior horn cell disease and autonomic disorder. *J Neurol*. 2013;260(1):301-302. doi:10.1007/s00415-012-6711-3.
59. Mehrpour M, Mohebi N, Motamed MR, Zamani F. Amyotrophic lateral sclerosis as a paraneoplastic manifestation in the neuroendocrine tumor of stomach: A case report. *Acta Med Iran*. 2013;51(10):724-726.
60. Younger DS, Graber J, Hayakawa-Yano Y, Parveen S, Frank M, Darnell RB. Ri/Nova gene-associated paraneoplastic subacute motor neuronopathy. *Muscle and Nerve*. 2013;47(4):617-

618. doi:10.1002/mus.23783.

61. Martinez L, Lamaze R, Clément-Duchêne C. Sclérose latérale primitive et adénocarcinome bronchique. Syndrome paranéoplasique ou association fortuite? *Rev Mal Respir*. 2013;30(3):227-230. doi:10.1016/j.rmr.2012.12.003.
62. Kacem I, Gnaichia C, Hizem Y, et al. *Paraneoplastic Motor Neuron Disease*. Vol e629. Elsevier B.V.; 2013. doi:10.1016/j.jns.2013.07.2201.
63. Geevasinga N, Burrell JR, Hibbert M, Vucic S, Ng K. C9ORF72 familial motor neuron disease - frontotemporal dementia associated with lung adenocarcinoma and anti-Ma2/Ta antibodies: a chance association? *Eur J Neurol*. 2014;21(4):31-33. doi:10.1111/ene.12347.
64. Spataro R, La Bella V. Paraneoplastic motor neuron disease associated with breast cancer. *Eur J Neurol*. 2014;21(1):5-6. doi:10.1111/ene.12295.
65. Diard-Detoeuf C, Dangoumau A, Limousin N, et al. Association of a paraneoplastic motor neuron disease with anti-Ri antibodies and a novel SOD1 I18del mutation. *J Neurol Sci*. 2014;337(1-2):212-214. doi:10.1016/j.jns.2013.11.025.
66. Struck AF, Salamat S, Waclawik AJ. Motor neuron disease with selective degeneration of anterior horn cells associated with non-hodgkin lymphoma. *J Clin Neuromuscul Dis*. 2014;16(2):83-89. doi:10.1097/CND.0000000000000056.
67. Pillainayagam C, Mella D, Johnson J, Woodman K. Unusual case of motor neuron disease as a paraneoplastic process (P4.230). *Neurology*. 2015;84(14 Supplement). http://www.neurology.org/content/84/14_Supplement/P4.230.abstract.
68. Verschueren A, Gallard J, Boucraut J, Honnorat J, Pouget J, Attarian S. Paraneoplastic subacute lower motor neuron syndrome associated with solid cancer. *J Neurol Sci*. 2015;358(1-2):413-416. doi:10.1016/j.jns.2015.08.014.
69. Erdener SE, Vural A, Temucin CM, et al. Brachial diparesis due to motor neuronopathy as one of the predominant presenting signs of occult small cell lung carcinoma. *Intern Med*. 2016;55(12):1641-1643. doi:10.2169/internalmedicine.55.4888.
70. Rosine N, Chretien P, Adam C, et al. Expression of Yo antigen in a prostatic adenocarcinoma. *Can J Neurol Sci*. 2017;44(2):221-223. doi:10.1080/13518040701205365.
71. Riahi A, Zaouali J, Bedoui I, Messelmani M, Mansour M, Mrissa R. Paraneoplastic motor neuron disease: A descriptive and prognostic study about five cases. *J Neurol Sci*. 2017;381:709. doi:10.1016/j.jns.2017.08.1996.
72. Suzuki Y, Aizawa H, Sakashita K, et al. Autopsy-proven case of paraneoplastic lower motor neuron disease with sensorimotor neuropathy due to Waldenström's macroglobulinemia.

*Neuropathology*. 2018;38(5):568-573. doi:10.1111/neup.12506.

73. Diamanti L, Quaquarini E, Berzero G, et al. Lower motor neuron syndrome in a patient with HER2-positive metastatic breast cancer: A case report and review of the literature. *Clin Neurol Neurosurg*. 2018;172(June):141-142. doi:10.1016/j.clineuro.2018.06.038.
74. Mélé N, Berzero G, Maisonobe T, et al. Motor neuron disease of paraneoplastic origin: a rare but treatable condition. *J Neurol*. 2018;265(7):1590-1599. doi:10.1007/s00415-018-8881-0.
75. Liu MG, Niu JW, Cui LY. A report on paraneoplastic motor neuron disease. *Chinese Med J*. 2019;132(6):719-722. doi:10.1097/CM9.0000000000000128.
76. Goodfellow J, Gorrie G, Leach V, Patel S, Mackay G. Cancer and motor neuron disease-causal or coincidental? Two contrasting cases. *Neurol Sci*. 2019;40(7):1461-1463. doi:https://doi.org/10.1007/s10072-019-03784-9.
77. Cheli M, Dinoto A, Ridolfi M, Sartori A, Stokelj D, Manganotti P. Motor neuron disease as a treatment responsive paraneoplastic neurological syndrome in patient with small cell lung cancer, anti-Hu antibodies and limbic encephalitis. *J Neurol Sci*. 2019;400:158-159. doi:10.1016/j.jns.2019.03.024.
78. La Bella V, Iannitto E, Cuffaro L, Spataro R. A rapidly progressive motor neuron disease associated to a natural killer cells leukaemia. *J Neurol Sci*. 2019;398:117-118. doi:10.1016/j.jns.2019.01.029.
79. Pinto WBV de R, Badia B de ML, Souza PVS de, Oliveira ASB, Silva LHL, Farias IB. Paraneoplastic motor neuronopathy and malignant acanthosis nigricans. *Arq Neuropsiquiatr*. 2019;77(7):527. doi:10.1590/0004-282X20190076.
80. Vogrig A, Joubert B, Maureille A, et al. Motor neuron involvement in anti-Ma2-associated paraneoplastic neurological syndrome. *J Neurol*. 2019;266(2):398-410. doi:10.1007/s00415-018-9143-x.
81. Jaffer M, Chung M, Sharda E, et al. Immunotherapy induced myasthenic-like syndrome in a metastatic melanoma patient with amyotrophic lateral sclerosis. *Clin Med Insights Oncol*. 2020;14:1179554920978024. doi:10.1177/1179554920978024.
82. Tolkovsky A, Kipervasser S, Fainmesser Y, Alcalay Y, Gadoth A. A paraneoplastic syndrome misdiagnosed as ALS: What are the red flags? A case report and review of the literature. *J Neuroimmunol*. 2021;358(May):577635. doi:10.1016/j.jneuroim.2021.577635.
83. Riku Y, Yoshida M, Tamura T, et al. Unexpected postmortem diagnoses in cases of clinically diagnosed amyotrophic lateral sclerosis. *Neuropathology*. 2021;41(6):457-467.

doi:10.1111/neup.12744.

84. Akan O, Baysal-Kirac L. Amyotrophic lateral sclerosis with coexisting cancer: a single-center study. *Acta Neurol Belg*. 2021;121(5):1123-1130. doi:10.1007/s13760-020-01337-y.
85. Shiba T, Sato M, Akisawa N, et al. A patient with mesenteric lymphoma who developed amyotrophic lateral sclerosis and sepsis. *Nippon Ronen Igakkai Zasshi*. 2021;58(3):476-481. doi:10.3143/geriatrics.58.476.
86. Kleinschmidt-Demasters BK, Marshall CB, Cykowski MD. Paraneoplastic Lower Motor Neuron Disease. *J Neuropathol Exp Neurol*. 2021;80(12):1125-1127. doi:10.1093/jnen/nlab078.
87. Rocha R, Ribeiro L, Correia F, Santos A, Martins J. Motor Neuron Disease associated with a neoplastic process: a review of cases. *Galicia Clínica*. 2021;82(2):87-89. doi:10.22546/61/2196.
88. Yang Z, He L, Ren M, et al. Paraneoplastic Amyotrophic Lateral Sclerosis: Case Series and Literature Review. *Brain Sci*. 2022;12(8):1053. doi:10.3390/brainsci12081053.
89. Echefu G, Arivazhagan S, Bandaru SS, Negulescu C. Paraneoplastic amyotrophic lateral sclerosis in a patient with pheochromocytoma. *Ann Case Reports*. 2023;8(01):1134. doi:10.29011/2574-7754.101134.
90. Vadell MR, F Miralles. "Flail arm syndrome" with anti-Hu antibodies. *Neurologia*. 2024;39(1):97-99. doi:10.1016/j.nrleng.2023.12.010.
91. Graus F, Vogrig A, Muñiz-Castrillo S, et al. Updated diagnostic criteria for paraneoplastic neurologic syndromes. *Neurol Neuroimmunol Neuroinflammation*. 2021;8(4):e1014. doi:10.1212/NXI.0000000000001014.
